# Climate change effects on ecosystem services: Disentangling drivers of mixed responses

**DOI:** 10.1371/journal.pone.0306017

**Published:** 2025-02-10

**Authors:** Marcy C. Delos, Ciara G. Johnson, Sarah R. Weiskopf, Janet A. Cushing

**Affiliations:** 1 Department of Environmental Science and Policy, George Mason University, Fairfax, Virginia, United States of America; 2 U.S. Geological Survey National Climate Adaptation Science Center, Reston, Virginia, United States of America; 3 U.S. Department of Energy, Oak Ridge Institute of Science Education, Oak Ridge, Tennessee, United States of America; UCL: University College London, UNITED KINGDOM OF GREAT BRITAIN AND NORTHERN IRELAND

## Abstract

Climate change is a pervasive hazard that impacts the supply and demand of ecosystem goods and services (EGS) that maintain human well-being. A recent review found that the impacts of climate change on EGS are sometimes mixed, posing challenges for managers who need to adapt to these changes. We expand on earlier work by exploring drivers of varying responses of EGS to climate within studies. We conducted a systematic review of English-language papers directly assessing climate change impacts on the supply, demand, or monetary value of ‘provisioning EGS’, ‘regulating EGS’, or ‘cultural EGS’. Ultimately, 44 papers published from December 2014 to March 2018 were analyzed. Nearly 66% of EGS were assessed for higher-income countries despite how lower-income countries disproportionately face negative climate impacts. Around 59% of observations or projections were mixed responses of EGS to climate change. Differences in climate impacts to EGS across space or climate scenarios were the most common causes of mixed responses, followed by mixed responses across time periods assessed. Disaggregating findings by drivers is valuable because mixed responses were often due to multiple drivers of variation. Carefully considering the decision context and desired outcome of a study will help select appropriate methodology to detect EGS variation. Although studies have often assessed relevant drivers of variation, assessing interactions of other sources of uncertainty and both climate and non-climate drivers may support more effective management decisions that holistically account for different values in the face of uncertainty.

## 1. Introduction

Ecosystems and biodiversity provide essential goods and services that maintain human well-being [1]. These goods and services have commonly been classified into provisioning services, such as food and water supply, regulating services, such as carbon sequestration and moderation of extreme events, and cultural services, such as tourism and cultural identity. Other ecosystem functions, including primary production and nutrient cycling, are critical for maintaining all ecosystem services [1].

While provisioning services related to agriculture and energy production have increased over the last 50 years, most of the services that nature provides to people have been declining [2–4]. Land use change has been the main driver of biodiversity and ecosystem service declines to date [3], but climate change effects have already been observed and will become an increasingly significant driver of change in the future [5]. Because ecosystem goods and services (EGS) are diverse, climate change may increase particular services while decreasing others. These interactions can be classified as trade-offs or synergies. For example, climate change may result in increased opportunities for leisure activities such as bicycling in North America [6]. In contrast, warmer winters with less snowfall have led to closures of ski resorts in New England [7]. Adding to this complexity, impacts to the same EGS can vary by location. For example, climate change is expected to reduce water yield provisioning for drinking water use in the Llobregat Basin of northeastern Spain [8], while increased precipitation in China’s Sancha River basin has increased water yield [9]. Even within individual studies, an EGS can show varied responses, which we classify as “mixed responses”.

A recent review of climate impacts on EGS [10] found that 24% of EGS analyses had mixed climate impacts, meaning that an EGS did not show a consistent directional response to climate change [10]. That review noted that variation in climate impacts across studies could differ by services, drivers, and assessment methods. We build upon this earlier work by exploring what drives varying climate responses within studies. To our knowledge, no quantitative literature review has focused on drivers, contexts, and methodologies that explain within-study variation in climate impacts on EGS.

The objective of this systematic review is to improve our understanding of what drives variable climate change impacts on EGS. Specifically, we examine whether and how these climate effects vary by space, time, climate scenarios, and other factors. Such findings (e.g., regarding patterns and research gaps) can support study designs that better incorporate relevant drivers of mixed responses, which can enable decisions to be more robust and flexible under variable and uncertain climate impacts. For example, optimal management approaches may differ for mixed EGS responses that are consistent across space, but decrease over time. To this end, our synthesis also highlights proactive approaches for managing EGS that vary across common drivers of mixed EGS responses.

## 2. Methods

### 2.1. Literature search and screening

We examined peer-reviewed journal literature that was published after the November 2014 publication period included in Runting et al. (2017) [10]. We included studies of any geographic setting or spatial scale. Since English was the primary language of our review team, we only screened English language papers, which could bias our conclusions [11–14]. We conducted our search using ProQuest Environmental Science Collection on March 15, 2018. Our keywords were “‘climate change’ AND ‘ecosystem service’ OR ‘ecosystem good’”. Because we sought a broadly representative sample of EGS, we did not include terms targeted to particular EGS sectors, climate drivers, or terms for frameworks closely tied to EGS.

To be included in our analysis, each assessment had to be a peer reviewed original research article (i.e., not a review paper) and had to meet the following criteria for incorporating climate change and EGS:

- Directly assessed climate impacts on provisioning, regulating, and/or cultural EGS supply, demand, or monetary value [15].
- Incorporated climate change.

This review was not registered and a protocol was not prepared, however see S1 Table 1 for further details on criteria for inclusion. Also see S2 Table for our PRISMA checklist. One reviewer independently screened each paper returned in our search. The list of included papers is available in S3 Table.

When we found multiple studies that relied on the same data, we included one paper that more closely fit our emphasis (e.g., focusing more on climate impacts on EGS than management impacts on EGS) to avoid double-counting [16]. Lastly, some studies did not mention an EGS-related term, however included EGS-focused models. These papers were included in our analysis (e.g., [17]).

### 2.2. Data-extraction

For each paper, we extracted data [18] related to the following categories (see S4 Table 1, adapted from Runting et al. [10]):

(i) Study area
(ii) Ecosystem services
(iii) Climate drivers
(iv) Heterogeneity in EGS Response
(v) Non-climate drivers
(vi) Uncertainty
(vii) Decision-making

We only retrieved data directly from our included papers; we did not contact authors for missing data. We extracted data from our included papers as follows: Each individual EGS in a paper was entered in a new row. If a paper assessed only a composite EGS value rather than separating out impacts on provisioning, regulating, or cultural EGS, we entered the service on a single row. EGS that were assessed in more than one habitat and/or time period were further subdivided into additional rows.

To ensure consistency in data extraction, we assigned a second reviewer for all papers. Reviewers worked independently, while any discrepancies between reviewers were discussed with the review team and resolved.

Problems with study methodology can lead to inaccurate results. We critically appraised each study before including the paper in our analysis. For instance, some studies presented findings as the impacts of climate change on ecosystem services, but due to the short length of the study (e.g., comparison over five years) were actually assessing the impact of climate variability on ecosystem services. We excluded these studies from our analysis.

### 2.3. Data analysis—Basic summary statistics

To analyze the characteristics, trends, and gaps within our papers, we calculated summary statistics based on our data-extraction questions. In most cases, summary statistics are based on the number of EGS assessed in specific habitats and time periods (e.g., carbon sequestration in both wetlands and oceans, over both observed and projected time periods). Where this is not the case (e.g., we present results at the paper-level), we note it in the results section. These results were visually displayed using basic Excel functions, Esri ArcGIS Pro, and R Statistical Software v4.1.3 [19].

## 3. Results

### 3.1. Context & characteristics of ecosystem goods and services

**Fig 1:**
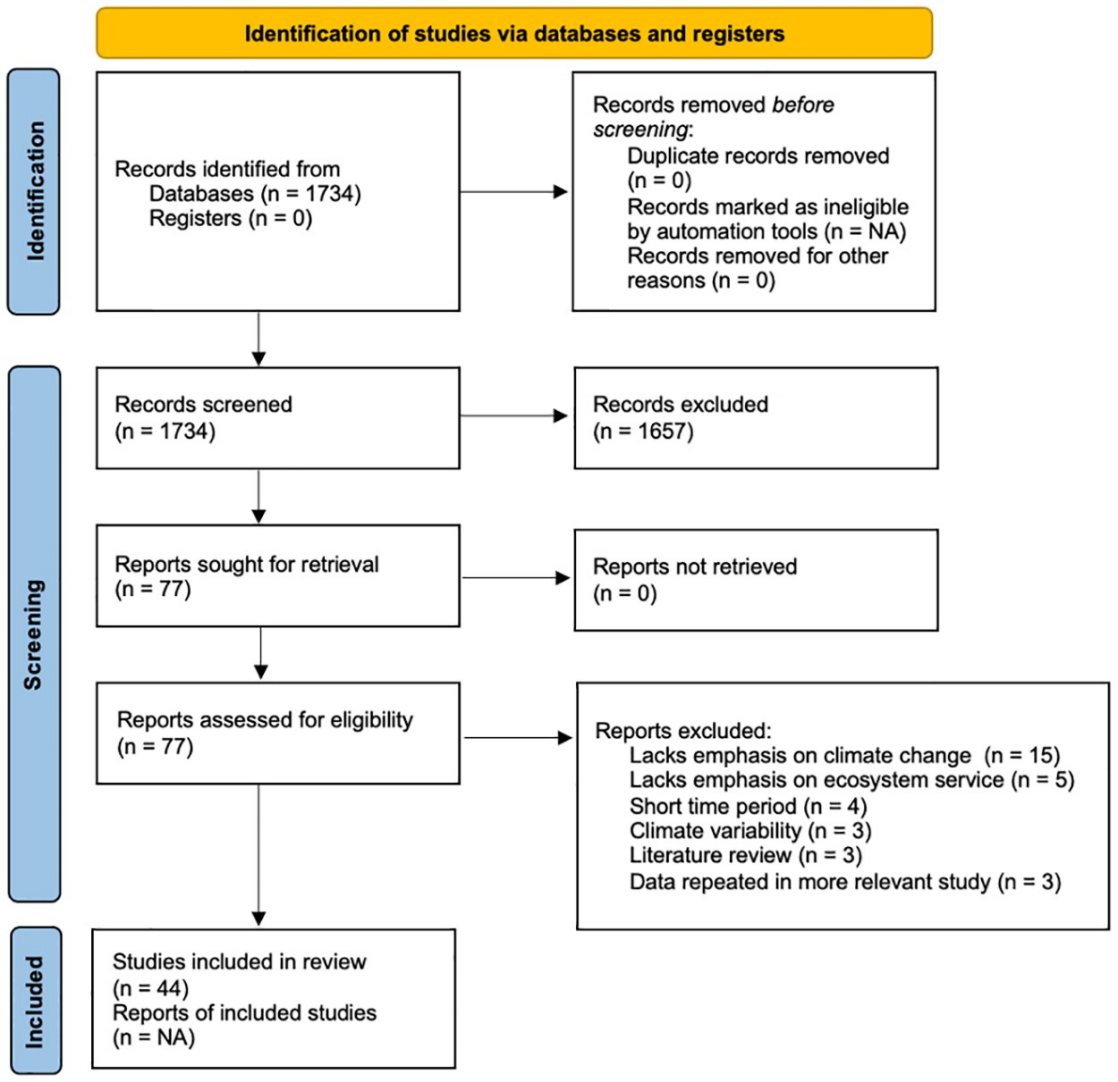
PRISMA flow diagram (2022). Literature search and selection process.

**Fig 2:**
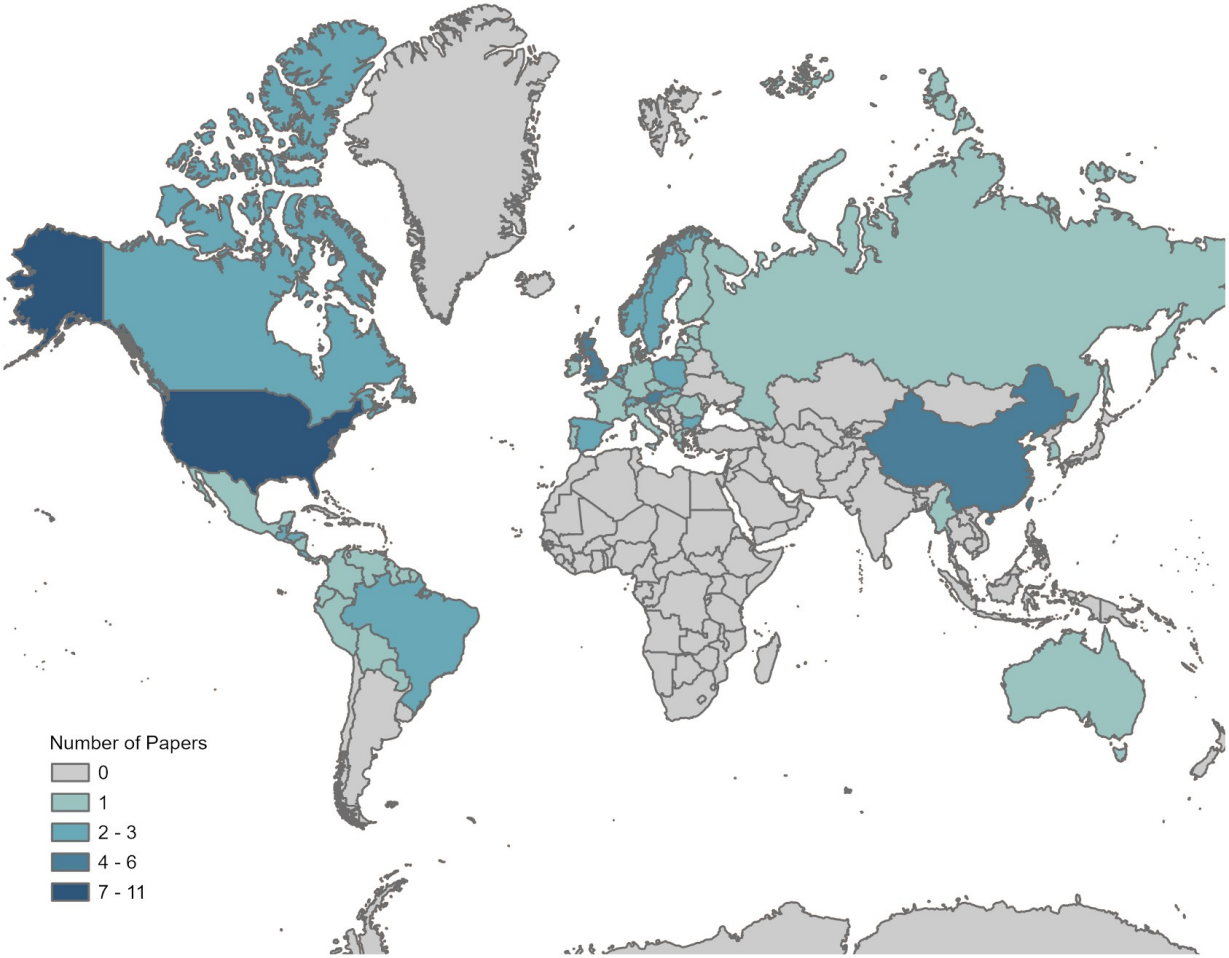
Map of number of studies by country at the paper-level. Darker blue indicates that a country was assessed by greater numbers of studies. Countries in grey were not assessed by any paper included in this review. French Guiana was counted as a separate country. These counts of countries are derived from 43 out of 44 total papers, since one paper focused on a global scale. Additionally, the study areas of three papers included major water bodies (e.g., marine ecosystems) nearby.

We included 44 papers in our final analysis, representing 55 countries. Our criteria for subdividing spreadsheet rows (see Methods) caused differences in total counts at the paper-level (44), EGS-level (100), level of EGS assessed in specific habitats and/or projected/observed periods (116), and EGS dimension for each EGS assessed in specific habitats and/or projected/observed periods (144). For detailed results of our literature screening process, see Fig 1. Regionally, Europe had the most papers, followed by North America and East Asia/Pacific regions (Fig 2). At the country-level, the United States had the highest number of papers (11), followed by China (6) (Fig 2). Across national income groupings [20], study areas were biased towards high-income countries. Sixty-six percent of study areas (61 study areas) were in high-income countries, 27% (25) in upper-middle-income countries, 7% (six) in lower middle-income countries, and none in low-income countries (aside from one paper that had a global scope). The spatial scale of each study varied, but regional scale (1,000–100,000 km^2^) was most commonly assessed (S1 Fig). Sixty-six percent of papers (29 papers) assessed EGS over only one spatial scale. EGS were most assessed in exclusively terrestrial ecosystems. Studies often included multiple types of habitats in a single analysis, although assessing strictly forest habitats was also particularly common (S2 Fig).

Regulating and provisioning services, especially service supply, dominated the EGS categories assessed (S3 Fig). Thirty-four percent of papers (15 papers) assessed only one regulating, provisioning, or cultural EGS. Even among the papers assessing multiple EGS, most papers assessed three or fewer. Two papers assessed multiple EGS in one composite value. Seventy percent of papers (31 papers) explicitly assessed interactions between EGS. Of the papers assessing interactions, nearly half (48%: 15 papers) found mixed interactions (both trade-offs and synergies). Strictly trade-offs or synergies were less frequent (each in 26%–i.e., 8 papers of those that clearly assessed interactions) (S4 Fig).

### 3.2. Impacts of climate change

Temperature and precipitation were the most frequently incorporated climate drivers of EGS changes (S5 Fig). Seventy-three percent of papers (32 papers) incorporated multiple climate drivers, usually assessed cumulatively. A majority of EGS were only assessed for projected rather than observed impacts of climate (S6 Fig). Climate impacts were usually assessed dynamically, incorporating change over multiple time points, with the majority between 55–105 years (full range is 15–200 years; S7 Fig). Almost one-fifth of papers (8 papers) had unclear timeframes or did not assess EGS over time (e.g., space-for-time substitution such as in Colloff et al., 2016 [21]).

**Fig 3:**
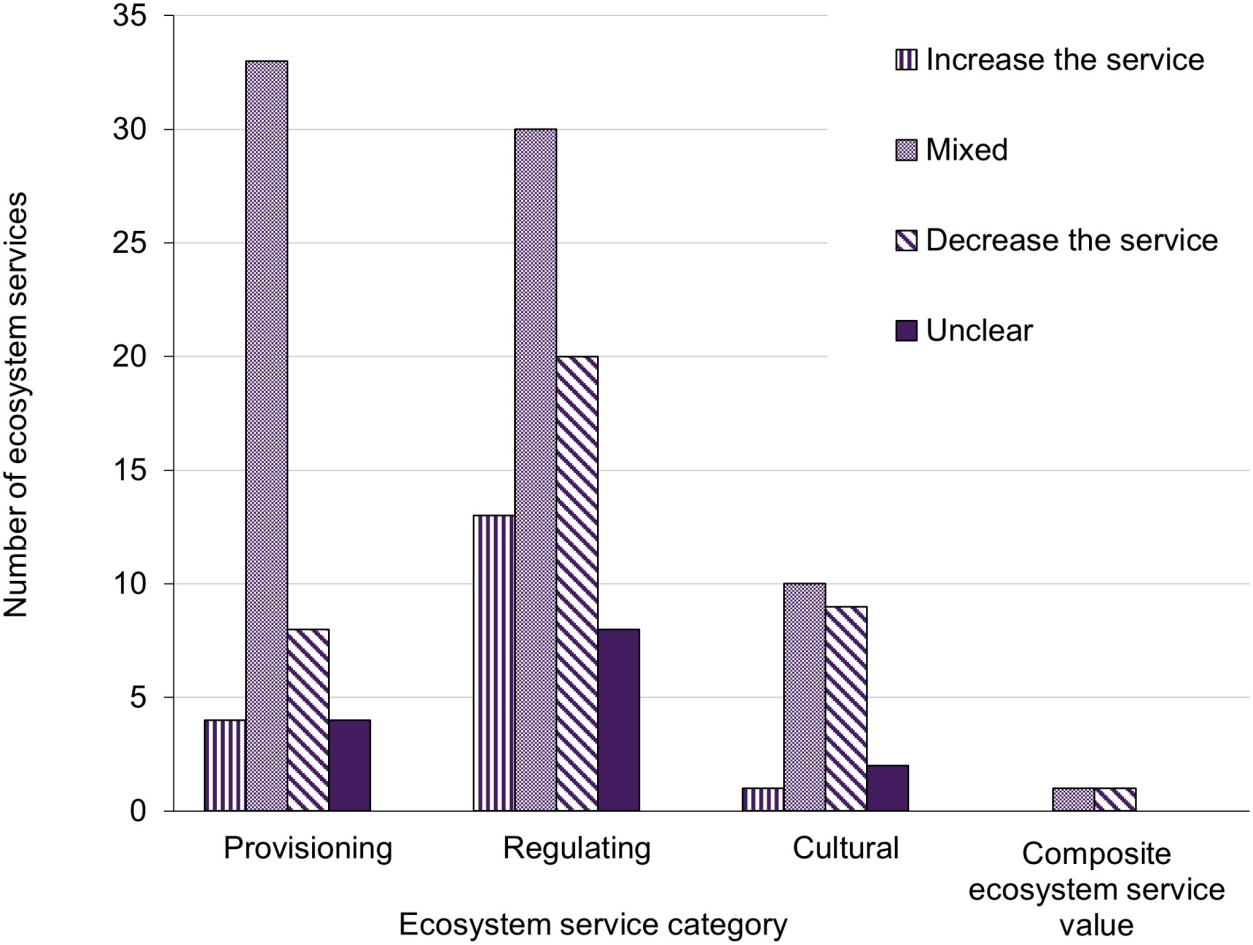
Direction of climate impacts on ecosystem service categories. A paper could assess climate impacts on supply, delivery/demand, and monetary value of the same ecosystem service, causing the total number of EGS assessed (144) to be greater than the total number of services assessed in specific habitats or time periods (116).

Many studies found mixed effects of climate change on EGS supply (59 out of 99 EGS; Fig 3). Of those finding directional responses, decreases in supply were slightly more common than increases (19 vs. 15, respectively). The impact of climate change on EGS delivery/demand was most frequently mixed or decreased (11 or 8, respectively). Lastly, climate change most often decreased EGS monetary value (11 out of 23).

### 3.3. Uncertainty

**Fig 4:**
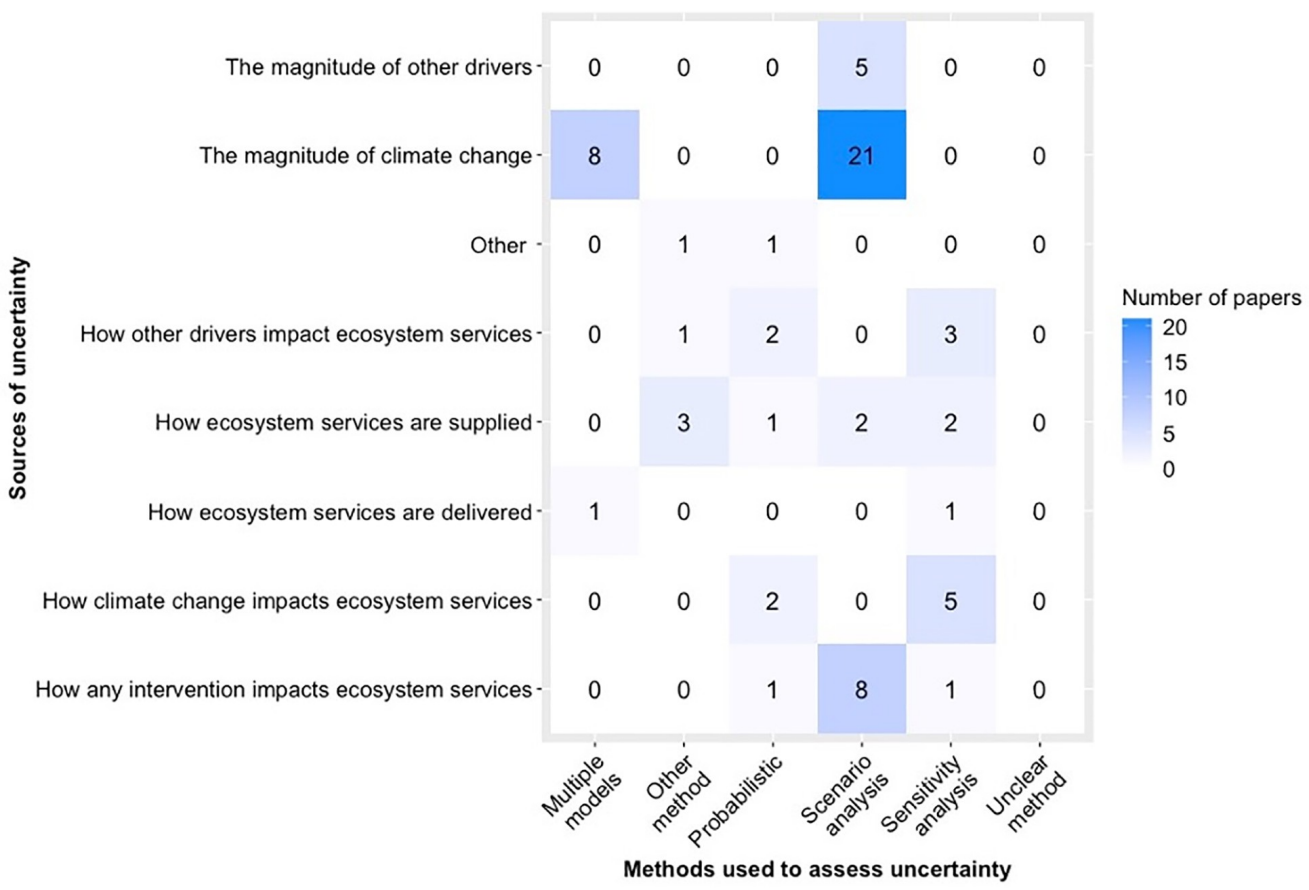
Sources and methods of assessing uncertainty or variability across papers. A paper could address multiple combinations of sources and methods of uncertainty, making the total number of source/method pairings (69) greater than the number of papers that explicitly assessed uncertainty (33 papers).

Uncertainty and/or variability was explicitly incorporated in 75% of papers (33 papers), and was at least mentioned in an additional 20% (9 papers). Most of those papers assessed uncertainties related to the magnitude of climate change. To assess uncertainty, scenario analysis was the most common method, followed by sensitivity analysis and the use of multiple models (Fig 4).

**Fig 5:**
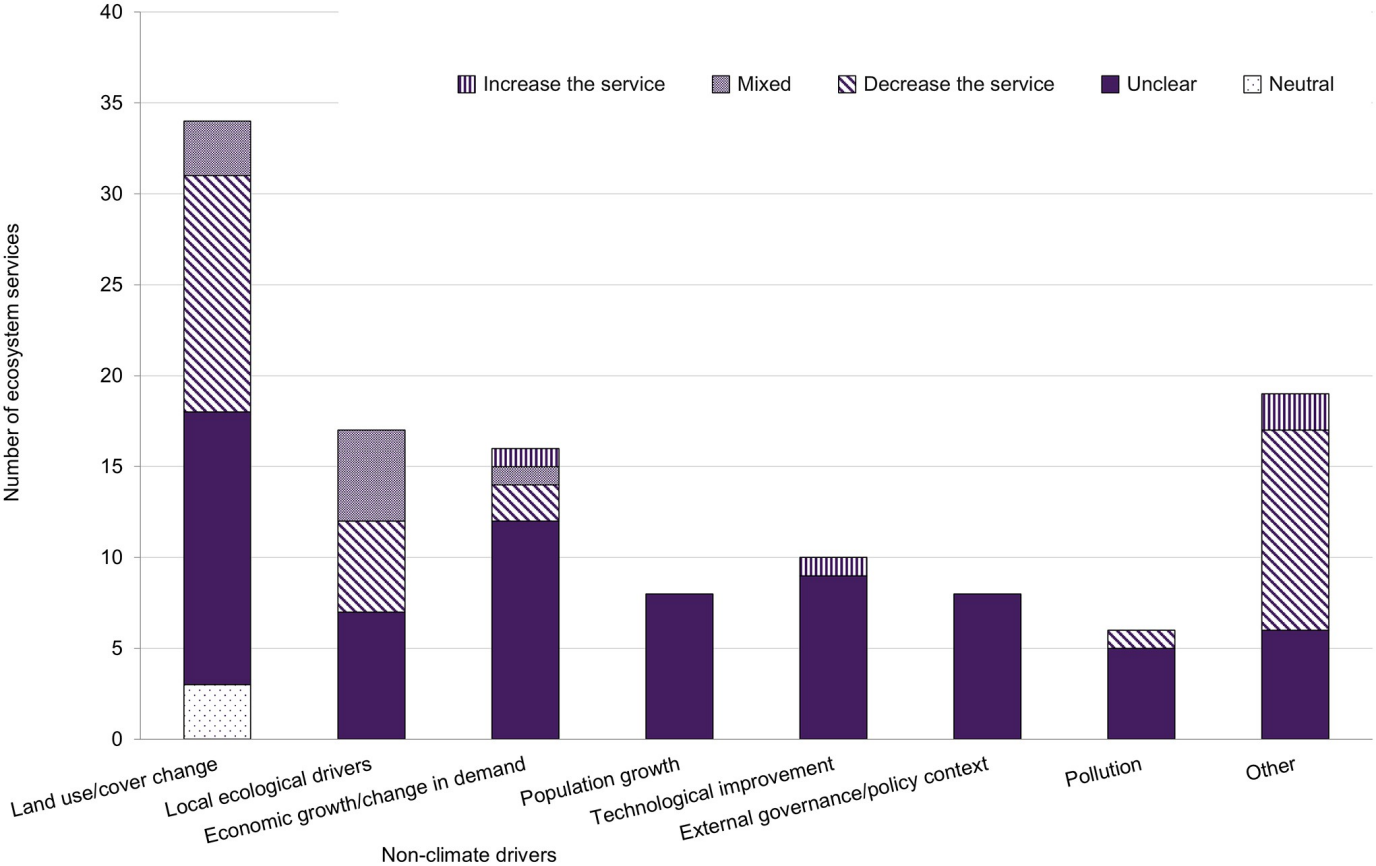
Impacts of non-climate drivers on ecosystem services. N = 118 impacts of non-climate drivers on ecosystem services (i.e., non-climate drivers matched to ecosystem service responses), which were assessed in specific habitats or time periods. A single paper’s ecosystem service in a specific habitat or time period could be assessed for multiple non-climate drivers. Such combinations are separately incorporated into the total number of impacts on ecosystem services presented. For this reason, the total number of pairings of non-climate drivers and ecosystem service responses (118 pairings) is greater than the number of ecosystem services for which both climate and non-climate impacts were assessed in specific habitats or time periods (59 ecosystem services).

Another source of uncertainty for climate change impacts on ecosystem services was the effect of non-climate drivers. The impact of non-climate drivers was unclear in almost 60% of EGS assessed (70 out of 118 EGS; Fig 5). Even when climate and non-climate drivers were assessed both individually and cumulatively, their interactions with EGS were often unclear.

### 3.4. Mixed responses to climate change

**Fig 6:**
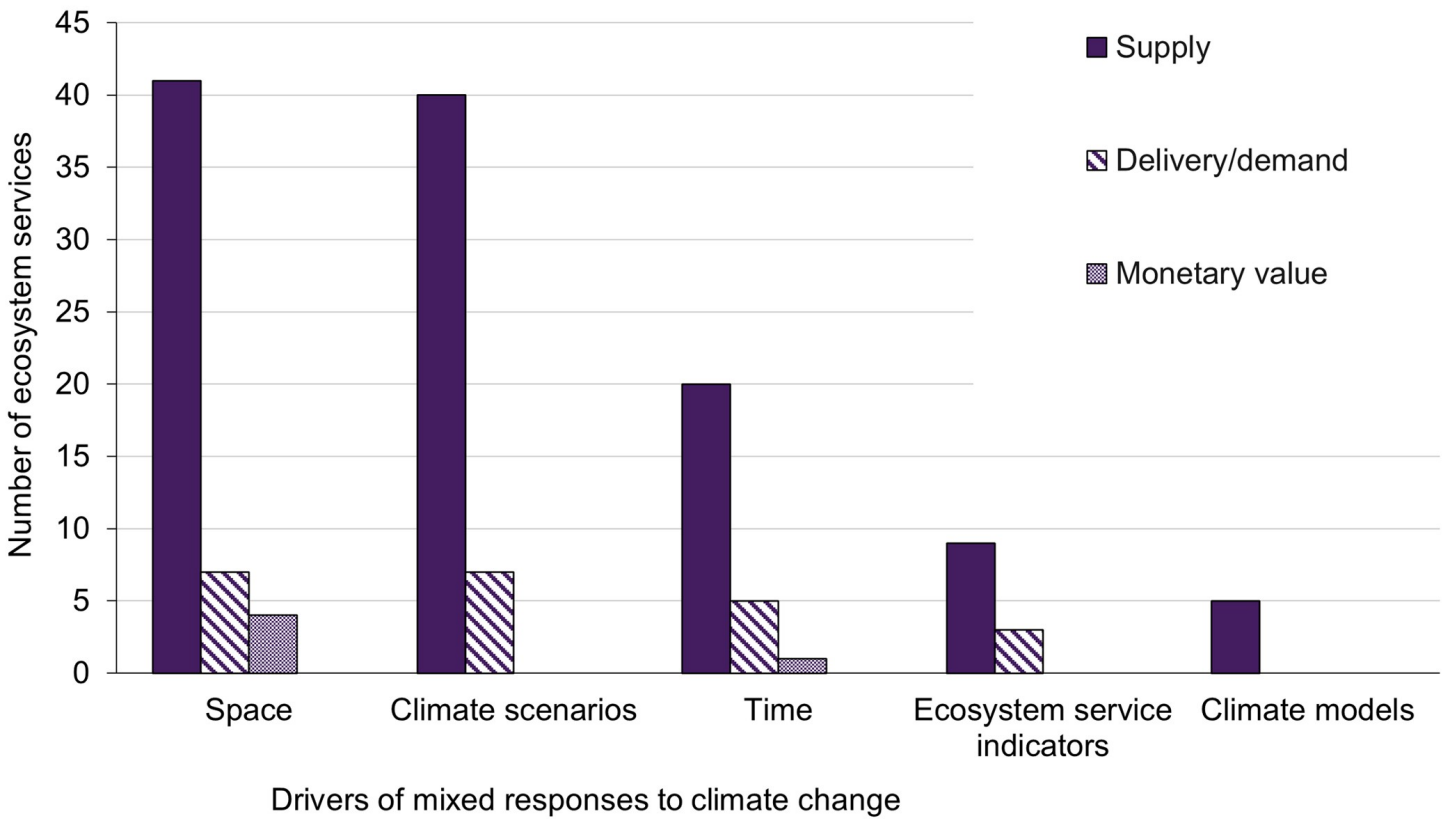
Number of ecosystem services that showed mixed climate change effects for particular drivers. N = 74 mixed EGS supply, delivery/demand, and monetary value responses to climate change. A single EGS response might show mixed climate effects due to multiple drivers. Therefore, the total number of mixed EGS response and driver combinations (142) is greater than the number of mixed EGS supply, delivery/demand, and monetary value responses (74).

Fifty-one percent of EGS responses to climate change were mixed (74 out of 144 EGS supply, delivery/demand, and monetary value responses). These mixed responses represented 60% of EGS supply (59 EGS), 50% of EGS delivery/demand (11 EGS), and 17% of EGS monetary value (4 EGS) among our reviewed studies. A mixed response indicates that the direction of climate impacts on EGS both increased, decreased, and/or showed no change depending on space (e.g., study area characteristics), EGS indicators, climate variables/scenarios/models used, or time frames assessed. Mixed responses were most often driven by differences across space (52 EGS: 70% of 74 mixed EGS responses), including elevation, region (e.g., political or biophysical categories such as counties or watersheds), or habitat characteristics (e.g., type, composition, or extent of vegetation; Fig 6). Varying intensity in climate scenarios (47 EGS: 64% of mixed EGS) and, to a lesser extent, fluctuations in EGS over time (26 EGS: 35% of mixed EGS) also drove mixed responses to climate change (Fig 6). Other study methodologies (e.g., using multiple EGS indicators, which comprised 12 EGS: 16% of mixed EGS) rarely drove mixed responses to climate change (Fig 6).

Mostly seen among the supply of EGS, mixed climate effects on EGS were often due to multiple, co-occurring drivers. Out of the 74 mixed EGS, 22 were mixed by only one driver, while 37 were mixed by two drivers and 15 were mixed by at least three drivers. Even when common drivers of variation were disaggregated (i.e., dividing EGS response by moderate or severe climate scenarios, or extracting EGS response at the end of the study’s time period), variable responses were still common. For example, when considering only the most severe climate scenario, 9% of EGS increased (3 EGS), 25% decreased (8 EGS), and 53% (17 EGS) still showed mixed responses; the remaining responses (4 EGS) were neutral. Moderate climate scenarios produced similar results, with only slightly fewer negative responses of EGS to climate change. Overall, mixed responses were most frequent when looking at the end of the study’s time period.

### 3.5. Decision-making

Nineteen out of 44 papers explicitly modeled or quantitatively assessed decision-making, 12 mentioned or discussed decision-making, and 13 did not consider decision-making. Of the papers explicitly modeling decision-making, 84% (16 papers) assessed multiple management objectives (e.g., freshwater EGS for consumption in addition to water-related recreation/tourism EGS [22]). Within the 16 papers that assessed both decision-making and uncertainty, 88% (14 papers) incorporated uncertainty implications for decision-making.

## 4. Discussion

As the Anthropocene produces novel drivers of ecosystem change, studies assessing EGS publish conflicting findings. We found that climate change can cause mixed effects on EGS in individual studies. A handful of study methodology factors are common drivers, including selection of spatial coverage, time scale, climate change scenarios, and EGS indicators. When we disaggregated findings by these drivers, only a few studies found consistent directional impacts of climate change on EGS [23–26]. Other studies remained mixed (for example, studies assessing EGS change over combinations of the following: multiple locations, time periods, varied-intensity climate change scenarios, or EGS indicators), highlighting the complexity of accurately predicting climate change impacts on EGS. Our study goes beyond previous work to identify the factors contributing to mixed responses, while summarizing the ability of recent climate-EGS literature to account for this variation and uncertainty.

### 4.1. Differences in effects across space

A study’s selection of study area, including the number and type of regions and habitats studied, can result in differences across space in EGS response to climate change. For example, Dunford et al. (2015) assessed EGS in Europe and found that the supply of raw materials varied depending on the sub-region studied [27]. Even differing elevations at one study site can be associated with contrasting outcomes [28]. Studies often assessed multiple habitats within one study region, and sometimes provided enough information to disaggregate the impacts by habitat type [29]. Studies that do not disaggregate impacts by habitat type may be sufficient for EGS that mainly occur in only one habitat type. Other more general EGS, such as recreation, could benefit from more habitat-specific results.

Simplifying assessments by only including one spatial scale often ignores how ecological and social processes operate [30, 31]. For example, a study on water supply in the Guizhou Province of China found declines across the entire region, but fluctuations at the finer watershed scale [32]. Each watershed had varied land-use compositions and rates of evaporation and precipitation [32]. Studies assessing multiple spatial scales (34%–i.e., 15 –of our papers) can capture more variation in EGS outcomes, leading to more effective adaptation actions [33]. It is important to note that each scale comes with its array of challenges; small scales consider important details, however add complexity and methodological difficulties [10, 34]. Large-scale, global assessments aid in intergovernmental collaboration, however are challenging to conduct because climate drivers and adaptation approaches vary at smaller scales, such as across regions [35–37]. Clearly communicating a study’s goals can help select the optimal spatial scale.

### 4.2. Differences in effects over time

Even outside the context of mixed climate effects, assessments of EGS more often focus on spatial patterns rather than temporal dynamics; EGS assessments frequently produce static snapshots of EGS at one point in time [38, 39]. These static studies may miss important temporal dynamics of supply, delivery, interactions, and unique drivers of change [40, 41]. Fluctuations in EGS response to climate change over time were one of the main drivers of mixed responses. Only dynamic assessments are able to uncover this variation, further emphasizing the usefulness of dynamic assessments that disaggregate multiple time periods.

We found that most papers assessed projected climate impacts on EGS. These often span multiple decades and/or centuries. Long study lengths can account for unanticipated impacts, especially those that are irreversible (e.g., species extinctions) [42]. For example, the supply of food may demonstrate a non-linear path over time while pest or disease events cause short-term disturbances in supply [38]. Mixed results can occur within these long-term projected studies. For example, under some climate and management scenarios in central Spain, forest timber production was higher in the climate change scenario compared to the no climate change baseline scenario, but was lower towards the end of the study period [24]. Despite challenges (e.g., lack of climate projections for short timescales [43]), studies that disaggregate over both near- and long- term are valuable: decision makers focused on near-term climate impacts can gain snapshots of relevant moments while also navigating long-term trade-offs. The study length may therefore depend on the decision under consideration and the risk tolerance of the decision-makers [42].

### 4.3. Differences in effects across climate change scenarios, models, and variables

In order to account for uncertainty in the magnitude of climate change, studies often include an array of climate scenarios, each varying in intensity (scenario analysis). Of these studies assessing multiple climate scenarios, some found that an EGS would increase under some scenarios, while decreasing under others. The type and number of climate variables included may have also influenced mixed responses. For example, Koenigstein et al. (2016) assessed food supply (i.e., fish stocks and minke whale populations) in both warming-only and warming and ocean acidification scenarios; scenarios including both showed more negative responses [44]. Although studies often incorporated multiple climate variables, they were often assessed cumulatively (e.g., as previously found in [10]), which may have contributed to why we rarely identified climate variables as drivers of mixed responses. For decision makers, the inclusion and disaggregation of a variety of climate scenarios and climate variables may cause confusion due to the large number of possible futures, but reduces uncertainty of decision makers. This transparency is key to new adaptive management frameworks, which can reduce overconfidence in policy actions and any resulting unintended outcomes [45–47].

### 4.4. Differences in effects across ecosystem service indicators

Some EGS cannot be quantified using only one indicator. This includes EGS with more complex ecosystem processes and dynamics. For example, climate-driven shifts in Swedish wetland plant community composition may increase nutrient retention under short-term but not long-term indicators [48]. For example, fast-growing species may increase nutrient uptake in the short-term, but rapid decomposition can prevent lasting increases in storage [48]. Cultural EGS such as recreation also can be measured using more than one indicator given the array of activities nature provides. For example, Colloff et al. (2016) assessed recreation in Australia using indicators for bushwalking, four-wheel driving, and camping [21]. These studies sometimes found varied responses among EGS indicators, though this was an infrequent driver of mixed EGS responses. By assessing important EGS indicators separately, managers can more easily evaluate trade-offs and synergies and prioritize management actions.

### 4.5. Other interactions (non-climate drivers)

Climate and non-climate drivers of change can work simultaneously [49] to disrupt EGS supply and demand. For example, ecosystems facing or recovering from disturbances (e.g., pest, disease, natural disaster) can be more sensitive to climate change [50]. Even if focused on climate adaptation, management actions can function as ecological stressors [51].

Impacts of non-climate drivers on EGS were frequently unclear, in some cases because papers bundled multiple non-climate drivers (e.g., in scenarios as previously characterized by [10]). Of the impacts that were clear, they frequently caused decreased or mixed EGS responses. Non-climate drivers impact EGS differently due to environmental factors, internal factors (e.g., species traits), and/or ecological interactions [52, 53]. The detection of these impacts can also vary by study methodology [52, 53]. These drivers may interact with climate impacts on EGS, therefore studies should consider analyses of non-climate and climate drivers separately and in conjunction with each other [10, 49]. An example of this methodology is disentangling how past climate and land-use change effects on water supply varied across space and time in the Tibetan Plateau, which provided evidence for the effectiveness of an ecological protection project [54]. Although the inclusion of non-climate drivers increases study complexity, integrated assessments can provide a more accurate measure of EGS change [10, 49, 55].

### 4.6. Interactions between ecosystem services

Assessing more than one EGS can help identify interactions between services (trade-offs and synergies), as well as track how climate effects or other impacts cascade across closely connected EGS [56]. Furthermore, sharing these EGS interactions with decision-makers may enhance their ability to deliver multiple EGS benefits or make transparent compromises [57]. Seventy percent of papers reviewed here assessed interactions between EGS, many of which found both trade-offs and synergies. Two papers in our review assessed multiple EGS through a composite EGS value [58, 59]. Although this approach does dramatically highlight the magnitude of EGS loss, it may obscure impacts on different EGS, EGS interactions, and beneficiary-specific access or valuation of EGS [57, 60–67].

### 4.7. Implications of uncertainty and mixed ecosystem service responses for decision-making

In response to uncertain effects of climate change and interacting drivers on EGS, more holistic, robust, and flexible decision-making is critical. Incorporating variation and uncertainty over drivers like space and time can help identify effective management interventions. Disentangling how past decisions and observed climate have affected EGS may help evaluate management effectiveness (e.g., at reducing harmful impacts or sustaining benefits among mixed climate effects), but may be especially challenging and/or undervalued given that most of our reviewed papers were projected rather than observed.

Most studies that assessed decision-making also assessed uncertainty (e.g., the magnitude of climate change or how climate impacts EGS). There are many types of uncertainty to consider, using various methods. However, both our studies and those from Runting et al. (2017) failed to include this variety [10]. Tools such as structured decision-making and value-of-information analyses [68–70], have been developed to assist managers in making decisions that are robust to uncertainty. Such tools could be used to identify which drivers of mixed or uncertain responses to climate change may be most influential to decisions [10, 71]. After initial decisions are made, managers can opt to re-evaluate the situation periodically to adapt to changing conditions; this is called adaptive management [72].

Periodic re-evaluation of decision actions, goals, and desired outcomes is key to the RAD (Resist-Accept-Direct) framework [73]. While conventional natural resource management practices thrive in a stable environment [74], RAD is an emerging management framework that addresses today’s complex, rapidly changing conditions [75]. Instead of exclusively resisting ecosystem change, the RAD framework presents managers with additional options to accept or direct change [75]. The RAD framework’s management approaches can be used sequentially (e.g., in response to threshold transitions of ecosystems, shifts in management goals or social values, or other forms of new information) or simultaneously (e.g., across space), so understanding how EGS will vary across these dimensions can inform decision making [75]. Locations where there is consistent EGS provisioning over time might be optimal for “resisting” change to maintain existing conditions, while knowing how services might shift over time can help managers plan when to “accept” changes or “direct” activities towards more favorable EGS [76]. For example, pollination supply is projected to shift from north to south, while remaining at high levels coastally in Brazil [77]. Thus, resisting changes in coastal areas, while directing agricultural shifts towards the south could make sense in this system. Another approach is to project spatial correlations in EGS demand and supply. Conservation actions across places with varied climate change impacts offer opportunities to safeguard EGS across space [e.g., 78].

Making decisions under frameworks like RAD requires identifying various potential outcomes, both desirable and unacceptable. This can be done by including a wide range of futures and clearly communicating uncertainty. Such approaches may be especially relevant in the EGS context, since services are shaped by both ecological and socio-economic conditions and management decisions.

### 4.8. Implications for research

To guide how future research can inform management approaches under varying and uncertain climate impacts, it is valuable to consider research gaps that are often persistent across the English language, peer-reviewed literature on EGS (S4 Table 2). For example, more frequent and stronger incorporation of climate impacts on delivery/demand (under-assessed among our reviewed studies and earlier literature [10]) may help to highlight what people value most, which would support decision-makers in evaluating management trade-offs over climate effects that differ across drivers like location and time.

While rarely assessed as drivers of mixed climate effects among our included studies, EGS indicators themselves could be more thoughtfully chosen in representing decision-relevant EGS dimensions like delivery/demand or monetary value. For example, indicators that could be incorporated and disaggregated to reveal negative climate impacts with important human rights implications, climate hazards [79] and labor conditions of EGS production deeply affect worker wellbeing, but are rarely captured in agricultural EGS value [80]. More broadly, disaggregation of EGS by beneficiary groups (e.g., Villarreal-Rosas et al. [71] regarding space, time, and socioeconomic dimensions) is under-studied [57, 81], and can reveal who benefits or is harmed through decisions to resist, accept, or direct change [82]. Differential benefits and harms can, for example, be examined through participatory approaches (e.g., pluralistic valuation) that meaningfully include marginalized communities [80, 83] and elevate traditional or local knowledges of climate impacts (e.g., [84]). In contrast to the prevalence of our reviewed papers that quantitatively assess climate impacts on EGS, it was rare to include stakeholder or rightsholder elicitation for linking climate-EGS impacts (S8 Fig) or assessing decision-making (S9 Fig); it may have been even more rare for such elicitation to acknowledge unequal power dynamics (e.g., discussed in [44, 80]), although such analysis was beyond the scope of our review. Novel research approaches which increase engagement between researchers and stakeholders or rightsholders, such as translational ecology, can tailor research methods and outcomes to meet management needs [85, 86].

It is also key to address how assessing a narrow, non-representative array of habitats and geographies limits our understanding [87] of varying climate effects and context-specific adaptation approaches, including locally-led innovation in the Global South [88]. While severely under-represented among our reviewed papers, lower-income countries endure disproportionate climate impacts and exacerbated vulnerabilities tied to international injustices (e.g., [89–95]), including appropriation of biophysical resources by high-income countries [96]. This underscores the decision-relevance of examining climate impacts on EGS in these areas, particularly because their communities often have high direct reliance on EGS [95, 97]. Such studies could, for example, illuminate ways to discern or reconceptualize priorities within mixed climate effects on EGS, such as through highlighting EGS decreases that hold no analogues or commensurability to EGS increases. These EGS decreases could include place-based losses that are understood through lived experiences, as well as through Indigenous and local knowledge systems [98]. Cultural EGS may be well-suited to capture such losses; however, our review found infrequent assessment of climate effects on cultural EGS (as with prior literature [10]), which is concerning coupled with other challenges like the tendency of economic valuation approaches to under-assess the losses faced by lower-income communities and countries (e.g., [90] regarding macroeconomics). Improved assessment of unavoidable losses may, for example, contribute to the growing knowledge of “losses and damages” (e.g., [93]) that justify global policy responses such as climate reparations (e.g., [92, 93]).

### 4.9. Limitations of our study

Our literature search only considered articles in English, introducing potential geographical bias in our results. English language bias may also restrict other features that our review examines for climate-EGS impacts, such as spatial scale (i.e., local scales are less likely to be published in English), habitat (i.e., landscape complexity [12], and endemic species or crop types of study areas [13, 14]). Examining English language papers allowed for reduced difficulties and potential errors in translating studies; however, future methodology can include other languages, evaluated by fluent speakers. Mirroring previous reviews (e.g., [10]), the imbalance of our reviewed study areas across geographies and national incomes may reflect not just English language bias, but also global inequities in research [11, 99–101]. These or other literature biases may have been reinforced through the broad scope of our search terms. Future reviews can, for example, focus search terms towards studies that address research gaps (e.g., continental- or regional-scale reviews like [102, 103]). The use of multiple article databases can aid in gathering literature across varied regions, disciplines, or sectors. A follow-up review can also include a formal assessment of risk of publication bias, as this was not included in this review. Ultimately, we did not contact authors to obtain data or clarify outstanding questions.

## 5. Conclusion

Given intensifying climate impacts on nature’s contributions to people, periodically synthesizing peer-reviewed evidence can inform proactive decision-making and research priorities. Our review highlighted key drivers of mixed climate impacts on EGS. A study’s choice of methodology can affect detection of mixed responses of EGS to climate change; common approaches–and associated drivers of mixed responses–include assessing multiple climate scenarios, spatial characteristics (e.g., habitat types, elevational gradients, and regions), and time periods. There are trade-offs in what results are presented (e.g., computational complexity and underlying value systems of the approaches incorporated); however, carefully considering the context (e.g., decision under consideration) and desired outcome of a study will help select appropriate methodology. Interactions between EGS with climate and non-climate drivers were also variable or unclear. Although studies have often incorporated relevant drivers of mixed climate effects, clearly assessing interactions of additional sources of uncertainty and both climate and non-climate drivers (i.e., long-standing research needs [10]) may lead to more effective management decisions that holistically account for different values in the face of uncertainty.

## Supporting information

S1 Table (1) Paper inclusion criteria. (2) Data-extraction questions and associated criteria.
(DOCX)

S2 Table PRISMA 2020 checklist for systematic reviews. (DOCX)

S3 Table List of included papers and full citations. (CSV)

S4 Table **(1) Categories of data-extraction questions.** Adapted from Runting et al., 2017 (Table 1). **(2) Research gaps in English language, journal-published assessments of climate impacts on ecosystem services**.
(DOCX)

S5 Table Spreadsheet of absolute totals (i.e., raw counts) for data represented in figures. (XLSX)

S1 Fig Total count of study spatial scale at the paper-level. Categories include micro (<1 km2), patch (1–100 km2), local (100–1,000 km2), regional (1,000–100,000 km2), national (100,000–1,000,000 km2), continental (1,000,000–100,000,000 km2), and global (>100,000,000 km2). The spatial scale recorded per study was the largest scale at which climate impacts on ecosystem services were assessed. N = 44 papers.
(TIF)

S2 Fig Frequency of habitat types by number of ecosystem services that were assessed in specific habitats or time periods (projected or observed climate change). N = 116 services assessed for climate impacts in habitats or time periods.
(TIF)

S3 Fig Frequency of the ecosystem service category (provisioning, regulating, cultural, or composite ecosystem service value) and EGS dimension (supply, delivery/demand, or monetary value) assessed across services in specific habitats and time periods. N = 144 EGS supply, delivery/demand, or monetary value responses to climate impacts in specific habitats and time periods.
(TIF)

S4 Fig Number of ecosystem service interaction types (synergy, trade-off, mixed, unclear) assessed per paper. Interactions could be between a provisioning, regulating, or cultural service and supporting services (e.g., biodiversity or habitat extent). A paper could assess multiple service interaction types (e.g., if the paper had multiple services with interactions each assessed). Consequently, the total number of ecosystem service interactions (33) summed to more than the total number of papers that clearly assessed interactions (31).
(TIF)

S5 Fig Frequency of climate change drivers assessed across papers. Since a paper could assess impacts of multiple climate drivers (an approach of 32 papers), the total number of climate drivers at the paper-level (80) is greater than the total number of papers (44).
(TIF)

S6 Fig Number of ecosystem services that were assessed for observed, projected, or both observed and projected impacts of climate change. N = 100 ecosystem services assessed within papers, without incorporating additional counts of services assessed in specific habitats or time periods.
(TIF)

S7 Fig Distribution of total number of years for assessing climate impacts on ecosystem services, including services that were assessed in distinct habitats and/or both projected and observed climate impacts. N = 94 services (81% of the total 116 services assessed by habitat or time period, since the total number of years within the remaining 22 services were either not assessed or unclear). Each range of years is inclusive of the bracketed numbers shown in the figure.
(TIF)

S8 Fig Frequency of methods used to assess climate impacts on ecosystem services, across papers. A paper could incorporate multiple methods to link climate impacts to services; therefore, the total number of methods at the paper-level (61) is greater than the total number of papers (44).
(TIF)

S9 Fig Frequency of method to assess decision-making across papers. A paper could incorporate multiple methods to assess decision-making. This caused the total number of methods to assess decision-making at the paper-level (24) to be greater than the total number of papers that assessed decision-making (19).
(TIF)
