# Supplementary material for: Climate change effects on ecosystem services: Disentangling drivers of mixed responses
Source: PLoS One. 2025 Feb 10;20(2):e0306017. doi: 10.1371/journal.pone.0306017 (PMC11809903; doi:10.1371/journal.pone.0306017)
Supplement: S1 Table — (1) Paper inclusion criteria. (2) Data-extraction questions and associated criteria. (DOCX) [file pone.0306017.s001.docx]

#### **S1 Tables: Criteria for data-extraction**

#### Abbreviations:

- Ecosystem good(s) and service(s): EGS
- Climate change: CC

**Full screening criteria for incorporating climate change and EGS**

To be included in our analysis, each assessment had to be a peer reviewed original research article (i.e., not a review paper) and had to meet the following criteria for incorporating climate change and EGS:

- Directly assessed climate impacts on provisioning, regulating, and/or cultural EGS supply, delivery/demand, or monetary value ([TEEB, 2010](http://doc.teebweb.org/wp-content/uploads/Study%20and%20Reports/Reports/Ecological%20and%20Economic%20Foundations/TEEB%20Ecological%20and%20Economic%20Foundations%20report/TEEB%20Foundations.pdf)):
- We included studies that presented climate change impacts on ecosystem disservices (Shackleton et al., 2016). For ecosystem disservices, we extracted the data to fit our ecosystem goods and services framing (e.g., if a paper discussed loss of freshwater as an ecosystem disservice, we extracted the data as freshwater provisioning) and noted that it was presented as a disservice.
- We included papers that assessed provisioning, regulating, or cultural EGS through proxy indicators (e.g., ecosystem structure elements like community composition). Although we allowed supporting services to represent proxies in that context (e.g., ecosystem functions like primary productivity as a proxy for carbon sequestration EGS), we excluded papers that focused on supporting services alone, as these services are often considered intermediate services rather than final services ([Boyd & Banzhaf, 2007](https://doi.org/10.1016/j.ecolecon.2007.01.002); Fu et al., 2011).
- Incorporated climate change:
  - Papers needed to examine climate impacts on EGS quantitatively or qualitatively.
  - We excluded papers that assessed how climatic variability affected EGS if this was not linked to potential climate change. For example, we excluded papers that assessed how EGS varied across a rainfall gradient without discussing how this change was related to projections of future change in the study area.
  - Although we included proxy measurements of projected climate impacts (e.g., substitutions of potential ecological responses across space for those that occur across time [[Blois et al., 2013](https://www.pnas.org/content/110/23/9374); [Banet & Trexler, 2013](https://doi.org/10.1371/journal.pone.0081025)]), we excluded papers that exclusively focused on how extreme weather events or climate adaptation interventions impact EGS.

Each paper’s abstract was reviewed to ensure its emphasis on climate and EGS. We also considered papers that did not directly mention climate or EGS in their abstract but did mention EGS-focused models (e.g., InVEST: Integrated Valuation of Ecosystem Services and Tradeoffs) or listed climate- or EGS-related keywords.

To avoid double-counting ([James et al., 2016](https://environmentalevidencejournal.biomedcentral.com/track/pdf/10.1186/s13750-016-0059-6)), when our search returned multiple papers that used the same data but otherwise fulfilled our inclusion criteria, we included the paper that most closely fit our emphasis (e.g., focusing more on climate impacts on EGS than management impacts on EGS). We opportunistically included one paper that contained climate-EGS assessment results from a model validation study returned in our search. The list of included papers is available in Appendix A.

#### **Table 1. Criteria for separating data into spreadsheet rows.**

| **Focus** | **Criteria for separation into rows** |
| --- | --- |
| **Each paper** | - Record each EGS of a paper on separate rows.   - However, if the same indicator is used to measure multiple EGS, list them in separate rows *only if* they are different EGS types (i.e., regulating, provisioning, cultural) or are assessed separately. |
| **Individual EGS within a paper** | - Within a single EGS of a paper, further subdivide responses into separate rows for any of these cases:   - “If a study assesses both observed and projected impacts on the same EGS.   - If climate impacts on the same EGS are separately assessed for different ecosystems. |

#### **Table 2. Data-extraction questions and associated criteria.**

| **Questions** | **Assumptions and criteria** |
| --- | --- |
| Q #1: Spatial scale of assessment  - Micro - Patch - Local - Regional - National - Continental - Global - Unclear | - *Largest* scale for which results (assessing CC impacts on EGS) are reported for a paper. - Ensure that scale chosen in this question is consistent with:   - Scale of assessing CC impacts on EGS (Q #18-#20)   - Scale of decision-making (whether decision-making is assessed is in Q #33), if applicable - Assign a spatial scale category by the specific size cut-off (km^2^ threshold) that fits study site(s), rather than the qualitative label associated with that category. - The size ranges for each category are as follows:   - Micro: <1 km^2^   - Patch: 1 – 100 km^2^   - Local: 100 – 1,000 km^2^   - Regional: 1,000 – 100,000 km^2^   - National: 100,000 – 1,000,000 km^2^   - Continental: 1,000,000 – 100,000,000 km^2^   - Global: > 100,000,000 km^2^ |
| Q #2: Single vs. multiple scales (for which climate impacts on EGS were assessed)?  - Single scale - Multiple scales - Unclear | - Scale(s) for which paper reports results of assessing CC impacts on EGS. - “Multiple scales” can include comparing multiple scales that fall into the same size classes of Q #1 (e.g., comparing CC-EGS impacts on “Regional”-scale counties vs. on the larger “Regional”-scale area that encompasses those counties). |
| Q #3: Location of assessment? (Countries and/or major water bodies that transcend national boundaries, if applicable) | - Country/countries and major water body/bodies where the study takes place. - If the study focuses on water body/bodies, also record the nearby countries that the paper also emphasizes (e.g., for having users who benefit from the water bodies and/or generate non-climate drivers – such as pollution – of EGS impacts). |
| Q #4: Type of ecosystem(s)?  - Terrestrial - Freshwater - Marine - Coastal - Unclear | - Record the ecosystem(s) where EGS are assessed for CC impacts (potentially in specific habitats or time periods of projected or observed CC).   - These should be the ecosystem(s) that the EGS are associated with – *not* the ecosystem(s) where the EGS benefits might also flow (even if the study assesses EGS delivery/demand). - “Coastal” includes ecosystems with an interface or gradient of freshwater/saltwater. |
| Q #5: Specific habitat  - Mixed   *Terrestrial:*   - Forest - Woodland & Shrubland - Grass & Rangeland - Desert - Tundra - Ice/Rock/Polar - Cultivated areas - Urban areas   *Freshwater:*   - Lakes & Rivers / other surface water - Inland wetlands - Groundwater   *Marine:*   - Open ocean - Coral reefs   *Coastal:*   - Seagrass/algae beds - Shelf sea - Brackish sea (e.g., Baltic Sea) - Estuaries - Shores (rocky shores and beaches) - Mangroves - Tidal marsh / Salt marsh - Unclear | - Focus on the particular habitat(s) (informed by [TEEB [2010]](http://www.teebweb.org/wp-content/uploads/2013/04/D0-Chapter-1-Integrating-the-ecological-and-economic-dimensions-in-biodiversity-and-ecosystem-service-valuation.pdf)) where EGS are assessed for CC impacts (potentially in specific habitats or time periods of projected or observed CC).   - Choose a single response per spreadsheet row – at the "resolution"/level of specificity that best matches how the study presents results.   - If the study assesses the same EGS for CC impacts in multiple habitats, record "Mixed" (i.e., reflecting mixed habitats). |
| Q #6: Which ecosystem service(s) are assessed? *Provisioning EGS:*  1. Food  2. Raw materials  3. Fresh water  4. Medicinal resources  *Regulating EGS:*  5. Local climate and air quality  6. Carbon sequestration and storage  7. Moderation of extreme events  8. Waste-water treatment  9. Erosion prevention and maintenance of soil fertility  10. Pollination  11. Biological control  *Cultural EGS:*  14. Recreation and mental and physical health / Tourism  16. Aesthetic appreciation and inspiration for culture, art and design / Spiritual experience and sense of place  18. Other cultural EGS  *If needed:*  19. Other ____ | - Record specific category of EGS that were directly assessed for CC impacts. - [TEEB (2010)](http://www.teebweb.org/wp-content/uploads/2013/04/D0-Chapter-1-Integrating-the-ecological-and-economic-dimensions-in-biodiversity-and-ecosystem-service-valuation.pdf) and [CICES (Haines-Young & Potschin, 2018)](https://cices.eu/content/uploads/sites/8/2018/01/Guidance-V51-01012018.pdf) informed our classification of EGS. - Cultural EGS includes existence or bequest values of biodiversity. |
| Q #7: Which indicator(s) are used to assess the ecosystem service(s)? | - Indicator(s): specific features used to assess an EGS (recorded at the service-level). - If multiple indicators (assessed in the context of CC) are incorporated for a single EGS category, list all indicators. |
| Q #8: If a food-related EGS is assessed, is it from a cultivated system (agricultural) and/or natural system (harvestable)?  - Agriculture-based - Harvestable (e.g., wild rice, berries, fish and game) - Both agriculture-based & harvestable - Unclear - NA | - Indicates whether the ecosystem service category “food” is from a cultivated or natural system. |
| Q #9: Are ecosystem disservices assessed?  - Yes - No - Unclear | - Indicates whether ecosystem disservice(s) were assessed at the service-level.   - Only record “yes” if the EGS of that row was described/assessed in the paper as a “disservice”. |
| Q #10: What position on the ecosystem service value chain is assessed?  - Supply - Delivery/demand - Monetary value | - Only record EGS value chain positions that were assessed for CC impacts. - If an EGS value chain position was driven only by decision-making beyond the study scale, group only within “other drivers” rather than in this column. E.g., changes in market price of EGS can be captured in the “other driver” of “Economic growth/demand increase”. - Categories   - Supply (potential) ([Tallis et al., 2012](https://doi.org/10.1525/bio.2012.62.11.7))   - Delivery/demand (actual) [(Tallis et al., 2012)](https://doi.org/10.1525/bio.2012.62.11.7)     - Distinguish potential delivery/demand (which should be assigned as “supply”) from actual delivery/demand.       - E.g., distinguish *potential* harvestable tree volume (supply) vs. actual harvestable tree volume (delivery/demand)   - Monetary value     - Do *not* list monetary value here if it is just an intermediate step (e.g., an input for an agent-based model), rather than reported as a final result under CC.     - Monetary value can be recorded both here and in decision-making approaches as long as CC impacts on monetary value are reported.     - Do *not* record if the monetary value was only assessed/reported under baseline CC. |
| Q #11: If monetary value is assessed, what valuation method is used?  - Market methods - Travel cost - Hedonic methods - Production approaches - Contingent valuation - Replacement cost - Avoidance cost - Benefit/value transfer - Other______ | - Categories, as defined by the citations below.   - Market methods     - - Market price approaches (Christie et al., 2012; [Turpie et al., 2003](https://reader.elsevier.com/reader/sd/pii/S0921800903001228?token=6E6773A61E3CC945169CF81E9C154140BFD3689A4F50973ABDFB7D5AEEE5CD0B166231D61F8E6F8982478F1A4F71E737); [Nijkamp](https://reader.elsevier.com/reader/sd/pii/S092180090800133X?token=9C6DDFDF7019A52EDD3F8F83AC677830B973FE7B12D3293AFF7DCC5F8D6824EF851E90AE459F136EDD85A55D2878EFAE) et al., 2006; [Nunes and van den Bergh, 2001](https://www.researchgate.net/publication/4840227_Economic_valuation_of_biodiversity_sense_or_nonsense/link/59e8b088a6fdccfe7f8eae47/download))     - Efficiency price / shadow price [(TEEB, 2010, Chapter 5)](http://africa.teebweb.org/wp-content/uploads/2013/04/D0-Chapter-5-The-economics-of-valuing-ecosystem-services-and-biodiversity.pdf)   - Travel cost (Clawson, 1959; [Hanley et al., 2002](https://www.researchgate.net/publication/5146536_Modelling_Recreation_Demand_Using_Choice_Experiments_Climbing_in_Scotland); Christie et al., 2012)   - Hedonic methods (Humavindu, 2003; Christie et al., 2012)   - Production approaches     - ([Amaza et al., 2006](https://citeseerx.ist.psu.edu/viewdoc/download?doi=10.1.1.835.2898&rep=rep1&type=pdf); Christie et al., 2012)   - Contingent valuation (Christie et al., 2012)   - Replacement cost (Christie et al., 2012)   - Avoidance cost ([TEEB, 2010, Chapter 5](http://www.teebweb.org/wp-content/uploads/2013/04/D0-Chapter-1-Integrating-the-ecological-and-economic-dimensions-in-biodiversity-and-ecosystem-service-valuation.pdf))   - Benefit/value transfer ([Wilson and Hoehn, 2006](https://reader.elsevier.com/reader/sd/pii/S0921800906004460?token=09E194A618624D731A24C8BC1A4C57EDC9EB590B1D054A6F0C35E81A423E1DCCA831015970585160467AB792B417EE23); Christie et al., 2012) |
| Q #12: Are interactions between services assessed (e.g., trade-offs)?  - Yes - No - Unclear - NA | - “Yes” indicates that interactions between EGS were assessed.   - The study must explicitly consider the interactions between the EGS of the spreadsheet row and other service(s) assessed in the paper. These interactions can be between provisioning/regulating/cultural services and supporting services.   - Terminology like “trade-offs” or “synergies” is not required to frame the interaction. However, interactions should be identified with at least some statement of relationship (e.g., “increase in EGS X and decrease in EGS Y”), in reference to the study’s results of how climate, non-climate drivers, and/or decision-making affect the EGS.   - Do not include interactions that are only discussed based on outside sources or mentioned as a limitation of the study. - “NA”: paper only assessed one provisioning/regulating/cultural EGS and did not assess its interactions with supporting services, or multiple EGS are assessed only as a composite. |
| Q #13: If interactions between services are assessed (e.g., trade-offs), identify the types of EGS interactions:  - Synergy - Trade-off - Mixed - Unclear - Other____ | - Categories   - Synergy     - Services “change in the same direction” simultaneously [(Dade et al., 2018)](https://link.springer.com/article/10.1007%2Fs13280-018-1127-7).     - Also includes Synergy and “Neutral”, where one EGS *increases* and the other EGS has *no* change in any definite direction.   - Trade-off     - Services change in “opposite direction” [(Dade et al., 2018)](https://link.springer.com/article/10.1007%2Fs13280-018-1127-7).     - Also includes Trade-off and “Neutral”, where one EGS *decreases* and the other EGS has *no* change in any definite direction.   - Mixed (Trade-offs and Synergies are present between services)     - E.g., over different:       - Time periods       - Parts of the study area       - CC scenarios       - Decision-making scenarios       - EGS indicators       - Non-climate drivers   - Unclear     - Unclear whether there are trade-off(s) or synerg(ies). |
| Q #14: What climate change variables are assessed?  - Change in temperature (for air or water temperature) - Change in precipitation - Change in extreme hydrologic events - Sea level rise - Ocean acidification - Carbon dioxide fertilization - Increased windspeed - Climate change proxy - Other_____ - Unclear | - Categories of climate aspects are informed by [IPCC (2014)](https://www.ipcc.ch/report/ar5/wg2/). - Record CC aspects that are explicitly modelled/measured (or discussed if no explicit modeling was conducted) for impacts on EGS, regardless of whether trend was ultimately found to be significant. - In studies that incorporate many climate inputs into models:   - Only record inputs that the study seems to intentionally vary or track for CC trends, since other climate inputs may have been held under baseline/stable conditions. |
| Q #15: Detailed description of climate variables that are mentioned as model inputs or otherwise incorporated into methods linking climate and ecosystem services. | - Service-level categories can include specific description of Q #14 climate variables as well as CO_2_ if its direct impact on EGS (e.g., through carbon dioxide fertilization) is assessed. |
| Q #16: Are these attributes of climate change assessed cumulatively, in isolation from each other, or both?  - Isolation - Cumulative - Both - Unclear - NA (only assessed one CC attribute) | - If multiple CC drivers are assessed, how they are assessed relative to each other. - Categories:   - In isolation from each other.   - Cumulatively     - Impacts of multiple CC aspects assessed together.   - Both (in isolation and cumulatively)     - Record if at least one climate variable is assessed in isolation and cumulatively.   - NA     - Only one CC aspect is assessed. |
| Q #17: Are climate impacts on ecosystem services observed or projected?  - Observed CC impacts on EGS - Projected CC impacts on EGS - Unclear | - Assessment of observed or projected impacts of climate on the ecosystem service. - Record proxy studies (e.g., space-for-time substitutions) as projected impacts. |
| Q #18-20: What is the impact of climate change on the ecosystem services studied? Respond in separate columns for EGS supply (Q #18), delivery/demand (Q #19), and monetary value (Q #20):   - Increase the EGS - Decrease the EGS - Neutral - Mixed (increase and decrease) - Unclear - NA (if paper’s EGS was not assessed for that particular position of the EGS value chain) | - Record the directions of CC impacts on EGS relative to baseline climate impacts on EGS. - Consider the direction(s) of CC impacts over the largest spatial scale for which CC-EGS results are reported - List “mixed” if any portion of the study area, any climate change variable or scenario assessed, any time period, or any EGS indicator has a response in a different direction. - If a study explicitly links CC to different directions of change between EGS rate (or monetary values per unit area) and EGS net impacts, record the direction of change of the net impacts.   - E.g., if a study reports an increase in the monetary value of coastal protection EGS per unit area of saltmarsh habitat, but a decrease in the overall monetary value of coastal protection EGS of the entire study area (from losses of saltmarsh habitat due to CC-related sea level rise): record the net decrease in monetary value as the direction of change. - In studies that assess CC impacts on multiple value chain positions (e.g., supply, delivery/demand) of the same EGS: record the CC impacts on the different EGS value chain positions in separate columns.   - If a study presents CC-EGS results for both demand and delivery (e.g., with separate directions of change depending on demand vs. delivery), we record CC impacts on delivery. |
| Q #20 (a): If the paper examines climate impacts on EGS monetary value, is the climate impact beneficial or harmful?  - Beneficial - Harmful - Neutral - Mixed (i.e., beneficial or harmful impacts) - Unclear - NA (e.g., CC impacts on the EGS monetary value were not assessed) | - Indicates whether the impact of CC on EGS monetary value was beneficial, harmful, mixed, etc. in the context of the EGS assessment (e.g., regarding the well-being of potential beneficiaries).   - These characterizations of beneficial or harmful provide nuance to a related data-extraction question (Q #20) that examines whether CC increased or decreased EGS monetary value. For example, increasing EGS monetary value might not have inherently beneficial implications; our responses to this data-extraction question could capture whether a study did (or did not) identify such implications. |
| Q #18-#20 (b): If applicable, what are the drivers of mixed climate impacts on EGS supply, delivery/demand, or monetary value? | - Drivers of mixed responses could include time, EGS indicator, space, habitat characteristics, region, and CC scenario.   - Following the driver, note in parentheses whether the driver was tied to a mixed response for EGS supply, delivery/demand, or monetary value.   - Space/habitat/region categories – examples:     - Space could include:       - Relative spatial positions (e.g., regarding pixels in maps of landscapes [[Mandle et al., 2021](https://www.nature.com/articles/s41893-020-00625-y.pdf)])       - Elevation     - Region could include: political or biophysical categories such as counties or watersheds, which could be overlaid within maps or presented solely as categorical data.     - Habitat characteristics could include: type, composition, or extent of vegetation. |
| Q #18-#20 (c, #1-#2): If there are mixed EGS responses under different climate scenarios, what are EGS responses under the most moderate climate scenario and the most severe climate scenario? | - If the study found mixed EGS responses under different CC scenarios and incorporated “moderate”/“severe” framings of CC scenarios, consider assessment of observed or projected impacts of CC on the EGS for the:   - Most moderate CC scenario only   - Most severe CC scenario only |
| Q #18-#20 (d): If there are mixed EGS responses under different time periods, what are EGS responses in the final time period assessed? | - Assessment of observed or projected impacts of CC on the EGS for the last time period only. |
| Q #18-#20 (e): Details regarding disaggregation of mixed EGS responses (i.e., drivers for why any EGS remained mixed across the disaggregated drivers, as well as whether EGS supply, delivery/demand, or monetary value were assessed). | - For each of the drivers by which we disaggregated mixed EGS responses:   - (i) What were the drivers for why EGS continued to show mixed responses (if applicable) even when disaggregated?   - (ii) What were EGS value chain components (i.e., supply, delivery/demand, monetary value) of EGS responses to CC? - Record the drivers by which we disaggregated mixed responses as “moderate scenario”, “severe scenario”, and “last period” (i.e., respectively indicating moderate climate scenario, severe climate scenario, and final time period of study).   - Matched to those disaggregated drivers, indicate the associated EGS response (mixed, increase, decrease, or neutral) if clear.   - For unclear EGS responses, do not specify the disaggregated driver unless the climate impact on EGS was both clear and unclear within that particular driver depending on EGS value chain component (e.g., within the final time period of a study, the climate impact was unclear for EGS delivery/demand but clearly increased for EGS supply). - Specify the relevant value chain component(s) in parentheses. - For the EGS responses that were still mixed after we performed disaggregation: use parentheses to indicate the driver(s) associated with that continued variation, matched to the relevant value chain component(s).   - Similar to what we described for a preceding data-extraction question, the drivers of that continued variation could include space (sometimes with “elevation” specified), time, EGS indicator, habitat characteristics, region, climate scenario, and climate model. |
| Q #21: If the paper includes analysis of observed climate impacts on ecosystem services, is the ecosystem service response significantly related to climate?  - Yes - No - Mixed - Unclear - NA | - If observed CC impacts are assessed: statistical significance of relationship between EGS response and CC. - Mixed: includes “yes” and “no” (or “unclear” and “no”) – e.g., depending on EGS indicator(s), CC aspect, time intervals within study period, and/or study area. - Unclear: includes when the spreadsheet row examines observed CC impacts on EGS, but statistical significance of that CC-EGS relationship is not assessed. |
| Q #22: What method is used to link climate change and ecosystem services?  - Statistical model (using field-based data) - Statistical model (using estimates) - Statistical model (using field experiment) - Process-based model (using field-based data) - Process-based model (using estimates) - Expert/Stakeholder Elicitation - Other ____ | - If multiple types of methods are used to link CC-EGS in the same study:   - Record the method(s) that *most directly* link CC and EGS. If it is unclear, record all methods. - Categories   - Regarding *inputs* for statistical, probabilistic, or process-based models:     - “Field-based data”       - Include:         - Any field data but at least some data is directly drawn from the study site(s).       - Exclude if:         - *Only* results from elicitation of experts, stakeholders, rightsholders.         - *All* field data is collected from outside the study area(s), but used to estimate the study area conditions in model parameters/inputs.         - Data from satellite images of study site (though aerial images are included as field-based data).     - Field experiment       - E.g., field studies in environments that represent proxies for CC (e.g. serving as space-for-time substitutions).   - Statistical model (e.g., see [Cuddington et al., 2013](https://doi.org/10.1890/ES12-00178.1)).   - Process-based model [(Buck-Sorlin, 2013](https://doi.org/10.1007/978-1-4419-9863-7_1545); [Cuddington et al., 2013](https://doi.org/10.1890/ES12-00178.1)). |
| Q #23: Was the method static, or does it assess changes over time (dynamic)?  - Static - Dynamic - Unclear - NA | - Assessment of climate impacts on ecosystem services over single or multiple time points (i.e., change over time), in addition to time point(s) of baseline climate effects.   - Categories refer to time point(s) for which the study explicitly presents CC-EGS result(s), even if the study mentions assessing additional time points. - Static: “assessing only one future or past time point in addition to the baseline” ([Runting et al., 2017](https://onlinelibrary.wiley.com/doi/abs/10.1111/gcb.13457)). - Dynamic: “assessing more than one future or past time points” ([Runting et al., 2017](https://onlinelibrary.wiley.com/doi/abs/10.1111/gcb.13457)). |
| Q #24: If applicable, list the time interval for which ecosystem services are assessed for climate impacts. | - Start year(s): First year of observations or projections.   - In assessments of observed climate impacts: if there was a first ‘census’ period, provide the year that represents the mid-point of that period. - End year(s): Last year of observations or projections.   - In assessments of observed climate impacts: if there was a last ‘census’ period, provide the year that represents the mid-point of that period. - If relevant, starting and/or ending time periods (i.e., ranges of years) may be recorded instead of individual years or mid-points. |
| Q #25: If applicable and clearly reported, list the total number of years assessed. | - Total years studied (last year-first year).   - In assessments of observed climate impacts: if census periods are used, take the mid-point of first and last period, and find *n* years between periods.   - In assessments of projected climate impacts: if the the study lists the ending period of a multi-year time interval as a decade (e.g., 2050s) rather than a single year, we record the midpoint of that decade (e.g., 2055 for 2050s) as the ending year. - NA   - Either the study did not take place over a specified time period or the number of years of the study was unclear. |
| Q #26: Are non-climate drivers considered and, if so, to what extent?  - Not considered - Mentioned/discussed - Explicitly modelled or otherwise quantitatively assessed | - Include decision-making as an “other driver” if the decision-making scale is larger (e.g., global policies [[Runting et al., 2017](https://onlinelibrary.wiley.com/doi/abs/10.1111/gcb.13457)]) or smaller (e.g., individual decisions) than the scale of the study area. - Categories   - “Not considered”   - “Mentioned/discussed”     - Mentions at least one “other driver” and the potential impact of that driver on the EGS, but does *not* need to explicitly state a direction of change of such impact(s).     - Exclude: discussion of starting conditions, especially those that remain constant over time.   - “Explicitly modelled or otherwise quantitatively assessed”     - See inclusion/exclusion criteria and categories in Q #27. |
| Q #27: If non‐climate driver(s) are assessed, list the specific type(s) of non‐climate driver(s).  - Land use/land cover change - Local ecological drivers - Resource extraction/degradation - Economic growth/change in demand - Population growth - Technological improvement - External governance/policy context - Pollution - Other _____ | - For other drivers that are “Explicitly modelled or otherwise quantitatively assessed”:   - Include:     - Varied by scenarios     - Assessed as a component of the model of EGS change   - Exclude:     - Starting conditions that remain constant over time, and are *not* varied through any of the above cases. - Drivers are only listed if their impact is presented separately. I.e., if multiple drivers contribute to a composite driver (e.g., population increase contributes to land use change), only list all drivers if the impact of each is discussed separately. |
| Q #28: What is the impact of the non-climate driver on the ecosystem service studied?  - Increase the EGS - Decrease the EGS - Neutral - Mixed (increased and decreased) - Unclear | - Describe the impact of non-climate drivers on EGS, if assessed in the study. Data are formatted as follows: “[driver] - [EGS response]”. In instances where multiple drivers are presented, each paired driver and response is separated by a comma. - Separate out non-climate driver categories when applicable.   - If it is difficult to isolate the potential impacts of individual non-climate drivers (e.g., in socio-economic scenarios that involve scenario-by-scenario changes across a suite of non-climate drivers, but only assess those drivers cumulatively): record “Unclear” as the impact for each non-climate driver. - In addition to the above criterion, apply Q #18-20 criteria here in the context of non-climate drivers, rather than climate drivers. |
| Q #29: How is the impact of the non-climate driver(s) assessed?  - In isolation from climate change impacts (only) - Cumulative impacts with climate change (only) - Both cumulative impacts and in isolation | - Describe how non-climate driver impacts are assessed relative to climate impacts. - Categories   - “In isolation from climate change impacts (only)”     - Impact of other drivers assessed separately from CC impacts only.   - “Cumulative impacts with climate change (only)”     - Impact of other drivers assessed cumulatively with CC impacts only.   - Both cumulative impacts and in isolation     - Includes disentangling the relative contributions of CC vs. other drivers or otherwise assessing impacts in isolation or cumulatively.     - To record “Both cumulative impacts and in isolation” in spreadsheet rows focused on projected CC impacts through scenario analysis, only the CC scenario needs to be evaluated in isolation (i.e., other drivers do not need to be assessed in isolation from CC). |
| Q #30: How does each driver interact with climate change?  - Synergistic - Antagonistic - Unclear - Mixed (Synergistic, Antagonistic) | - If non-climate and climate driver(s) are assessed both cumulatively and in isolation (per [Runting et al., 2017](https://onlinelibrary.wiley.com/doi/abs/10.1111/gcb.13457)): interaction(s) of non-climate driver(s) with climate change. Responses are matched to specific non-climate driver(s). In instances where multiple drivers are presented, each paired driver and response is separated by a comma. - Categories (informed by Brown et al., 2013)   - “Synergistic” (climate and non-climate drivers have the same direction of impacts)   - “Antagonistic” “Synergistic” (climate and non-climate drivers have opposite directions of impacts)   - “Mixed”     - Includes: “Synergistic” and “Antagonistic”     - E.g., different interactions over different areas (e.g., elevations) in space.   - “Unclear” (challenging to characterize the interactions)   - “NA” (non-climate drivers were not assessed) |
| Q #31: Is uncertainty (or variability) considered and, if so, to what extent?  - Uncertainty not considered - Uncertainty mentioned/discussed - Uncertainty explicitly incorporated - Unclear | - Use of “uncertainty” terminology in our review includes uncertainty *and/or* variability. - Categories   - “Uncertainty mentioned/discussed”     - Includes: a paper explicitly comparing only one scenario of change to a baseline scenario, as well as a paper discussing uncertainty without explicitly assessing any uncertainty source(s)/method(s).   - “Uncertainty explicitly incorporated”     - Includes: quantitative (e.g., modeling) or qualitative assessment of uncertainty (e.g., qualitative scenarios or expert/stakeholder/rightsholder elicitation), as well as when results of uncertainty assessment are not reported but the paper mentions performing a particular source/method of uncertainty assessment (e.g., through performing sensitivity analysis for how climate impacts EGS). - See Q #32 for included categories of sources and methods of uncertainty. |
| Q #32: For explicitly-assessed uncertainty:What is the source of the uncertainty, and what methods are used to incorporate it in the assessment?*Sources:*The magnitude of climate changeThe magnitude of other driversHow climate change impacts ecosystem servicesHow other drivers impact ecosystem servicesHow any intervention (e.g. management) impacts ecosystem servicesHow ecosystem services are suppliedHow ecosystem services are delivered/demandedOther (specify)*Methods:*Scenario analysis (comparison of different, internally consistent, sets of assumptions about the future)Multiple models (assessment is carried out using different models of the same system)Sensitivity analysis (varying parameters of the analysis)ProbabilisticUnclear method _____Other method (specify) | - If a paper assesses uncertainty (or variability), note the source(s) of uncertainty and method(s) to incorporate it. Paper-level source-method combinations are recorded. E.g., if a paper used both multiple climate models and multiple emissions scenarios to assess uncertainty in the magnitude of climate change, record both “The magnitude of climate change - Multiple models” and “The magnitude of climate change - Scenario analysis”. - Categories:   - Sources     - The magnitude of climate change     - The magnitude of other drivers     - How climate change impacts ecosystem services       - Can include uncertainty in how CC impacts EGS due to variability in weather.     - How other drivers impact ecosystem services     - How any intervention (e.g., management) impacts ecosystem services       - Can include uncertainty in the magnitude of any intervention that, in turn, impacts EGS (does necessarily have to focus on the "how"/mechanism of that impact).     - How ecosystem services are supplied       - Uncertainty in factors that affect EGS supply, *if* those factors are not closely related to uncertainty in CC, non-CC drivers, and/or decision-making.     - How ecosystem services are delivered/demanded     - Other (e.g., scale of measurement)      - - Methods (Polasky et al. [2011], Yousefpour et al. [2012], Refsgaard et al. [2007], and others)     - Scenario analysis (comparison of different, internally consistent, sets of assumptions about the future)       - Requires at least two scenarios vs. baseline.       - Can include running a “single”, average value of different scenarios.     - Multiple models (assessment is carried out using different models of the same system)       - Examining differences from running multiple, independent models (or across averaged values from multiple models).     - Sensitivity analysis (varying parameters of the analysis)     - Probabilistic       - Examples:       - Monte Carlo analysis, as well as other approaches that involve many model runs under random seeds or use random processes to generate variability in a model component.       - Bayesian (e.g., Bayesian belief networks, which is a graphical model that represents a set of variables and their conditional dependencies**)**     - Unclear method     - Other method |
| Q #33: Is decision-making (i.e., actions, policies, or other interventions) considered and, if so, to what extent?  - Not considered - Mentioned/discussed - Explicitly modelled or otherwise quantitatively assessed - Unclear | - The spatial scale of decision-making (in contrast to Q #26 regarding non-climate drivers) approximately matches the largest spatial scale at which climate impacts are reported (Q #1, per [Runting et al., 2017](https://onlinelibrary.wiley.com/doi/abs/10.1111/gcb.13457)). - Categories   - “Mentioned/discussed”     - Includes: Any discussion of implications for decision-making.       - Can record “mentioned/discussed” for both the “non-climate drivers” and “decision-making” attributes if the mentioned large-scale policy is also discussed in a decision-making context.   - “Explicitly modelled or otherwise quantitatively assessed”     - Record for decision-making that is assessed through quantitative measurement/modelling or qualitative approaches like descriptive scenarios or expert/stakeholder/rightsholder elicitation. The impact of the decision can be either direct or indirect.     - For further detail, also see:       - Categories of decision-making in Q #36       - Methods for assessing decision-making in Q #35 |
| Q #34: If decision-making is explicitly assessed:Are single or multiple objectives of decision-making explicitly assessed?  - Single - Multiple - Unclear - NA (decision-making was not explicitly assessed) | - If a paper assesses decision-making: indicate whether decision-making incorporated single or multiple objectives.   - Objectives: any category(ies) of services (provisioning/cultural/regulating EGS or supporting services) that were a focus of decision-making that is explicitly assessed under CC. |
| Q #35: What method is used to model or assess the action, policy, or interventions?  - Process-based model (scenarios) - Statistical model (scenarios) - Qualitative model (scenarios) - Expert/Stakeholder elicitation / narrative - Cost-benefit / other economic analysis - Optimization - Adaptive management - Bayesian belief network (scenarios) - Structured decision-making (e.g., multi-criteria decision analysis) - Other method - Unclear method | - If a paper assesses decision-making: record method(s) used to assess the decision(s). Multiple methods could be listed for a single paper.   - If a paper primarily uses composite method(s) to assess decision-making (e.g., a method that encompasses other method(s) similar to how adaptive management can include optimization): record the overarching method(s) unless the other methods are discussed separately. - Categories   - Process-based model (scenarios)   - Statistical model (scenarios)   - Qualitative model (scenarios)   - Expert / Stakeholder elicitation / narrative   - Cost-benefit / other economic analysis     - Cost-benefit analysis     - Cost-effectiveness analysis     - Replacement cost or restoration cost   - Optimization   - Adaptive management [(Epanchin-Niell et al., 2018)](https://doi.org/10.3133/cir1439)   - Bayesian belief network (scenarios [[Smith et al., n.d.])](http://openness.hugin.com/huginprog/documentation/WP3_Method_Guidelines_BBNs_050314.pdf).   - Structured decision-making ([Keeney, 1992](https://www.hup.harvard.edu/catalog.php?isbn=9780674931985&content=toc); [Hammond et al., 1999](https://journals.sagepub.com/doi/10.1177/0272989X9901900315); [Gregory et al.. 2012](https://www.wiley.com/en-us/Structured+Decision+Making%3A+A+Practical+Guide+to+Environmental+Management+Choices-p-9781444333411); [Robinson et al., 2016](https://esajournals.onlinelibrary.wiley.com/doi/full/10.1002/ecs2.1613); Gregory & Keeney 2002; Runge, 2011; [Saarikoski et al., 2016](http://www.openness-project.eu/sites/default/files/SP_MCDA.pdf)) |
| Q #36: What category do these actions, policies or other interventions fall into?  - Testing site-based management actions - Allocating management actions across space - Allocating a range of land uses (land use zoning) - Allocating protected areas - Specific legislation - Financial / market solutions - Awareness raising / education / coordination - Technological solutions - Other _____ - Unclear | - If a paper assesses decision-making: record type(s) of decision(s). - If the same decision falls into multiple categories: record the category or the most specific component (e.g., of legislation) that acts upon EGS. - Explicitly identifying priority areas for a particular type of decision-making also qualifies, if the paper shows clear criteria for assigning the type of decision-making. - Categories   - Testing site-based management actions     - One location/site.     - Site often has socially/politically defined boundaries. E.g., consider a wildlife refuge to be one site, rather than having areas of different elevations within that wildlife refuge represent different locations/sites.   - Allocating management actions across space     - Multiple locations/sites (e.g., states, parks).   - Allocating a range of land uses (land use zoning)     - Can include zoning for *one*, rather than multiple, uses.   - Allocating protected areas (see ed. [Dudley, 2008](https://portals.iucn.org/library/sites/library/files/documents/PAG-021.pdf) for definition of protected area)     - “Allocating” can describe, for example, (re)drawing boundaries of a protected area.   - Specific legislation   - Financial / market solutions     - E.g., payment for ecosystem services schemes, subsidies, levies, reverse auction, creation of new markets   - Awareness raising / education / coordination     - E.g., for coordination: sectors – that otherwise compete for EGS – work together for planning   - Technological solutions     - E.g., improved harvesting or irrigation technology |
| Q #37: Are the implications of uncertainty for decision-making considered and, if so, to what extent?  - Not considered - Mentioned/discussed - Explicitly incorporated or otherwise quantitatively assessed - Unclear | - Categories   - “Mentioned/discussed”     - Includes:       - Mention or discuss the implications of uncertainty for decision-making, but do not explicitly assess the consequences for specific actions.       - Explicitly compare only one scenario of change to a baseline scenario and discuss implications for decision-making.   - “Explicitly incorporated or otherwise quantitatively assessed”     - Includes but is not limited to:       - Assessing uncertainty in Q #25 regarding “How any intervention (e.g. management) impacts ecosystem services”       - Assessing the intervention(s) under different CC and/or non-climate scenarios (with more than one scenario considered relative to “baseline”).       - Assessing decision-making via adaptive management. |

#### **References**

Amaza, P. S., Bila, Y., & Iheanacho, A. C. (2006). Food Crop Production in West Africa: Empirical Evidence from. *Journal of Agriculture and Rural Development in the Tropics and Subtropics*, *107*(2), 139–147.

Banet, A. I., & Trexler, J. C. (2013). Space-for-Time Substitution Works in Everglades Ecological Forecasting Models. *PLoS ONE*, *8*(11), e81025. https://doi.org/10.1371/journal.pone.0081025

Blois, J. L., Williams, J. W., Fitzpatrick, M. C., Jackson, S. T., & Ferrier, S. (2013). Space can substitute for time in predicting climate-change effects on biodiversity. *Proceedings of the National Academy of Sciences*, *110*(23), 9374–9379. https://doi.org/10.1073/pnas.1220228110 ‌

Boyd, J., & Banzhaf, S. (2007). What are ecosystem services? The need for standardized environmental accounting units. *Ecological Economics*, *63*(2), 616–626. https://doi.org/10.1016/j.ecolecon.2007.01.002

Buck-Sorlin, G. (2013). Process-based Model. In W. Dubitzky, O. Wolkenhauer, K.-H. Cho, & H. Yokota (Eds.), *Encyclopedia of Systems Biology* (pp. 1755–1755). Springer. <https://doi.org/10.1007/978-1-4419-9863-7_1545>

Christie, M., Fazey, I., Cooper, R., Hyde, T., & Kenter, J. O. (2012). An evaluation of monetary and non-monetary techniques for assessing the importance of biodiversity and ecosystem services to people in countries with developing economies. *Ecological Economics*, *83*, 67–78. <https://doi.org/10.1016/j.ecolecon.2012.08.012>

Clawson, M. (1959). *Method of measuring the demand for and value of outdoor recreation*. Resources for the Future, Inc.

Cuddington, K., Fortin, M.-J., Gerber, L. R., Hastings, A., Liebhold, A., O’Connor, M., & Ray, C. (2013). Process-based models are required to manage ecological systems in a changing world. *Ecosphere*, *4*(2), art20. <https://doi.org/10.1890/ES12-00178.1>

Dade, M. C., Mitchell, M. G. E., McAlpine, C. A., & Rhodes, J. R. (2019). Assessing ecosystem service trade-offs and synergies: The need for a more mechanistic approach. *Ambio*, *48*(10), 1116–1128. <https://doi.org/10.1007/s13280-018-1127-7>

de Groot, R., Fisher, B., & Christie, M. (2010). Integrating the ecological and economic dimensions in biodiversity and ecosystem service valuation. In *The Economics of Ecosystems and Biodiversity: The Ecological and Economic Foundations* [TEEB].

Dudley, N. (Ed.). (2008). *Guidelines for applying protected area management categories*. International Union of Concerned Scientists. <https://doi.org/10.2305/IUCN.CH.2008.PAPS.2.en>

Epanchin-Niell, R. S., Boyd, J. W., Macauley, M. K., Scarlett, L., Shapiro, C. D., & WIlliams, B. K. (2018). *Integrating Adaptive Management and Ecosystem Services Concepts To Improve Natural Resource Management: Challenges and Opportunities* (Circular No. 1439; Circular, p. 62). U.S. Department of the Interior U.S. Geological Survey.

Fu, B.-J., Su, C.-H., Wei, Y.-P., Willett, I. R., Lü, Y.-H., & Liu, G.-H. (2010). Double counting in ecosystem services valuation: causes and countermeasures. *Ecological Research*, *26*(1), 1–14. https://doi.org/10.1007/s11284-010-0766-3 ‌

Gregory, R., Failing, L., Harstone, M., Long, G., McDaniels, T., & Ohlson, D. (2012). *Structured Decision Making: A Practical Guide to Environmental Management Choices*. Wiley-Blackwell.

Gregory, R. S., & Keeney, R. L. (2002). Making Smarter Environmental Management Decisions. *Journal of the American Water Resources Association*, *38*(6), 1601–1612. <https://doi.org/10.1111/j.1752-1688.2002.tb04367.x>

Haines-Young, R., & Potschin, M. (2018). *Common International Classification of Ecosystem Services (CICES) V5.1 and Guidance on the Application of the Revised Structure* (p. 53). Fabis Consulting Ltd.

Hammond, J. S., Keeney, R. S., & Raiffa, H. (1999). *Smart Choices: A Practical Guide to Making Better Decisions*. Harvard Business School Press.

Hanley, N., Wright, R. E., & Koop, G. (2002). Modelling Recreation Demand Using Choice Experiments: Climbing in Scotland. *Environmental and Resource Economics*, *22*(3), 449–466. <https://doi.org/10.1023/A:1016077425039>

Humavindu, M. N., & Stage, J. (2003). Hedonic pricing in Windhoek townships. *Environment and Development Economics*, *8*(2), 391–404. <https://doi.org/10.1017/S1355770X0300202>

IPCC, 2014: *Climate Change 2014: Impacts, Adaptation, and Vulnerability. Part A: Global and Sectoral Aspects. Contribution of Working Group II to the Fifth Assessment Report of the Intergovernmental Panel on Climate Change* [Field, C. B., Barros, V. R., Dokken, D. J., Mach, K. J., Mastrandrea, M. D., Bilir, T. E., Chatterjee, M., Ebi, K. L., Estrada, Y. O., Genova, R. C., Girma, B., Kissel, E. S., Levy, A. N., MacCracken, S., Mastrandrea, P. R., and White, L. L. (eds.)]. Cambridge University Press, 1132 pp.

James, K. L., Randall, N. P., & Haddaway, N. R. (2016). A methodology for systematic mapping in environmental sciences. *Environmental Evidence*, *5*(1). https://doi.org/10.1186/s13750-016-0059-6 ‌

Keeney, R. L. (1996). *Value-Focused Thinking A Path to Creative Decisionmaking*. Harvard University Press.

Mandle, L., Shields-Estrada, A., Chaplin-Kramer, R., Mitchell, M. G., Bremer, L. L., Gourevitch, J. D., Hawthorne, P., Johnson, J. A., Robinson, B. E., Smith, J. R., Sonter, L. J., Verutes, G. M., Vogl, A. L., Daily, G. C., & Ricketts, T. H. (2020). Increasing decision relevance of Ecosystem Service Science. *Nature Sustainability*, *4*(2), 161–169. https://doi.org/10.1038/s41893-020-00625-y

Nijkamp, P., Vindigni, G., & Nunes, P. A. L. D. (2008). Economic valuation of biodiversity: A comparative study. *Ecological Economics*, *67*(2), 217–231. <https://doi.org/10.1016/j.ecolecon.2008.03.003>

Nunes, P. A. L. D., & van den Bergh, J. C. J. M. (2001). Economic valuation of biodiversity: Sense or nonsense? *Ecological Economics*, *39*(2), 203–222. <https://doi.org/10.1016/S0921-8009(01)00233-6>

Pascual, U., Fisher, B., Muradian, R., Brander, L., Gómez-Baggethun, E., Martín-López, B., & Verma, M. (2010). The economics of valuing ecosystem services and biodiversity. In *The Economics of Ecosystems and Biodiversity: The Ecological and Economic Foundations* [TEEB].

Polasky, S., Carpenter, S. R., Folke, C., & Keeler, B. (2011). Decision-making under great uncertainty: Environmental management in an era of global change. *Trends in Ecology & Evolution*, *26*(8), 398–404. <https://doi.org/10.1016/j.tree.2011.04.007>

Refsgaard, J. C., van der Sluijs, J. P., Højberg, A. L., & Vanrolleghem, P. A. (2007). Uncertainty in the environmental modelling process – A framework and guidance. *Environmental Modelling & Software*, *22*(11), 1543–1556. <https://doi.org/10.1016/j.envsoft.2007.02.004>

Runge, M. C. (2011). An Introduction to Adaptive Management for Threatened and Endangered Species. *Journal of Fish and Wildlife Management*, *2*(2), 220–233. <https://doi.org/10.3996/082011-JFWM-045>

Robinson, K. F., Fuller, A. K., Hurst, J. E., Swift, B. L., Kirsch, A., Farquhar, J., Decker, D. J., & Siemer, W. F. (2016). Structured decision making as a framework for large‐scale wildlife harvest management decisions. *Ecosphere*, *7*(12). <https://doi.org/10.1002/ecs2.1613>

Runting, R. K., Bryan, B. A., Dee, L. E., Maseyk, F. J. F., Mandle, L., Hamel, P., Wilson, K. A., Yetka, K., Possingham, H. P., & Rhodes, J. R. (2017). Incorporating climate change into ecosystem service assessments and decisions: A review. *Global Change Biology*, *23*(1), 28–41. <https://doi.org/10.1111/gcb.13457>

Saarikoski, H., Barton, D. N., Mustajoki, J., Keune, H., Gomez-Baggethun, E., & Langemeyer, J. (n.d.). Multi-criteria decision analysis (MCDA) in ecosystem service valuation. *OpenNESS*, 6.

Shackleton, C. M., Ruwanza, S., Sinasson Sanni, G. K., Bennett, S., De Lacy, P., Modipa, R., Mtati, N., Sachikonye, M., & Thondhlana, G. (2016). Unpacking Pandora’s Box: Understanding and Categorising Ecosystem Disservices for Environmental Management and Human Wellbeing. *Ecosystems*, *19*(4), 587–600. https://doi.org/10.1007/s10021-015-9952-z

Smith, R., Madsen, A. L., Haines-Young, R., & Barton, D. (n.d.). WP3 Methodological Guidelines for Bayesian Belief Networks. *OpenNESS*, 9.

Tallis, H., Mooney, H., Andelman, S., Balvanera, P., Cramer, W., Karp, D., Polasky, S., Reyers, B., Ricketts, T., Running, S., Thonicke, K., Tietjen, B., & Walz, A. (2012). A Global System for Monitoring Ecosystem Service Change. *BioScience*, *62*(11), 977–986. <https://doi.org/10.1525/bio.2012.62.11.7>

Turpie, J. K. (2003). The existence value of biodiversity in South Africa: How interest, experience, knowledge, income and perceived level of threat influence local willingness to pay. *Ecological Economics*, *46*(2), 199–216. <https://doi.org/10.1016/S0921-8009(03)00122-8>

Wilson, M. A., & Hoehn, J. P. (2006). Valuing environmental goods and services using benefit transfer: The state-of-the art and science. *Ecological Economics*, *60*(2), 335–342. <https://doi.org/10.1016/j.ecolecon.2006.08.015>

Yousefpour, R., Jacobsen, J. B., Thorsen, B. J., Meilby, H., Hanewinkel, M., & Oehler, K. (2012). A review of decision-making approaches to handle uncertainty and risk in adaptive forest management under climate change. *Annals of Forest Science*, *69*(1), 1–15. <https://doi.org/10.1007/s13595-011-0153-4>
