# Supplementary material for: Climate change effects on ecosystem services: Disentangling drivers of mixed responses
Source: PLoS One. 2025 Feb 10;20(2):e0306017. doi: 10.1371/journal.pone.0306017 (PMC11809903; doi:10.1371/journal.pone.0306017)
Supplement: S4 Table — (1) Categories of data-extraction questions. Adapted from Runting et al., 2017 (Table 1). (2) Research gaps in English language, journal-published assessments of climate impacts on ecosystem services. (DOCX) [file pone.0306017.s004.docx]

**S4 Tables: Data extraction questions and research gaps**

**Table 1.** Categories of our data-extraction questions. This table is adapted from [Runting et al. (2017, Table 1)](https://doi.org/10.1111/gcb.13457).

| Categories | No. | Question |
| --- | --- | --- |
| (i) Study area | 1 | Spatial scale of assessment |
|  | 2 | Single vs. multiple scales (for which climate impacts on ecosystem service are assessed) |
|  | 3 | Location of assessment |
|  | 4 | Ecosystem type(s) |
|  | 5 | Habitat type(s) |
| (ii) Ecosystem services | 6 | Ecosystem service assessed |
|  | 7 | Indicator(s) used to assess the ecosystem service |
|  | 8 | If a food-related ecosystem service is assessed: Cultivated system (agricultural) and/or natural system (harvestable) |
|  | 9 | Ecosystem disservices assessed |
|  | 10 | Position(s) on the ecosystem service value chain: Supply (potential), delivery/demand (actual), and/or monetary value |
|  | 11 | If monetary value is assessed: Valuation method(s) used |
|  | 12 | Interactions between services assessed (e.g., trade-offs) |
|  | 13 | If interactions between services are assessed: Type(s) of ecosystem service interactions |
| (iii) Climate drivers | 14 | Climate change variables assessed |
|  | 15 | Detailed description of climate variables |
|  | 16 | Were climate change variables assessed cumulatively, in isolation from each other, or both? |
|  | 17 | Were climate change impacts observed or projected? |
|  | 18 | Impact of climate change on ecosystem service supply |
|  | 19 | Impact of climate change on ecosystem service delivery/demand |
|  | 20 | Impact of climate change on ecosystem service monetary value |
|  | 20 (a) | If climate impacts on ecosystem service monetary value are assessed: Beneficial or harmful impacts? |
|  | 18-20 (b) | If applicable: Driver(s) of mixed climate impacts on ecosystem service supply, delivery/demand, or monetary value |
|  | 18-20 (c) | If the ecosystem service is mixed across climate scenarios: Direction(s) of climate impacts on the ecosystem service under moderate and severe climate scenarios only |
|  | 18-20 (d) | If the ecosystem service is mixed across time periods: Direction(s) of climate impacts on the ecosystem service in the last time period of the study |
|  | 18-20 (e) | If applicable: Details regarding disaggregation of mixed ecosystem service responses (i.e., drivers for why any ecosystem service remained mixed across disaggregated drivers, as well as whether ecosystem service supply, delivery/demand, or monetary value were assessed) |
|  | 21 | If the paper includes analysis of observed climate impacts on ecosystem services: Significance of ecosystem service response to climate |
|  | 22 | Method used to link climate change and ecosystem services |
|  | 23 | Is method static or does it assess changes over time (i.e., dynamic)? |
|  | 24 | If applicable: Time interval for which ecosystem services are assessed for climate impacts |
|  | 25 | If applicable: Total number of years assessed |
| (iv) Non-climate drivers | 26 | Were non-climate drivers considered? |
|  | 27 | If non‐climate driver(s) are incorporated: Type(s) of drivers (e.g., land use change, economic growth, and other socio-economic variables) |
|  | 28 | Impact of the non‐climate driver(s) on the ecosystem service studied |
|  | 29 | How the impact of non-climate driver(s) is assessed (i.e., in isolation from climate change, cumulatively, or both) |
|  | 30 | How non-climate drivers interact with climate change |
| (v) Uncertainty | 31 | Was uncertainty and/or variability considered? |
|  | 32 | Source(s) of uncertainty and method(s) used to incorporate it in the assessment |
| (vi) Decision-making | 33 | Consideration of decision-making (i.e., actions, policies, or other interventions) |
|  | 34 | If decision-making is explicitly assessed: Single or multiple objectives of decision-making |
|  | 35 | Method(s) used to model or assess the action, policy, or interventions |
|  | 36 | Type(s) of policies or other interventions |
|  | 37 | Were implications of uncertainty for decision-making considered? |

**Table 2**. Research gaps in English language, journal-published assessments of climate impacts on ecosystem services. Many research gaps that our review identified are similar to those of previously published literature (e.g., see [Runting et al., 2017)](https://doi.org/10.1111/gcb.13457).

| Categories | Research gaps |
| --- | --- |
| (i) Study area | Areas and community priorities in underrepresented regions (e.g., Africa, Latin America, the Caribbean, South Asia), particularly in low‐income and lower middle‐income countries that face disproportionate climate impacts and other global inequities ([Chausson et al., 2020](https://onlinelibrary.wiley.com/doi/full/10.1111/gcb.15310); [World Bank, 2020](https://datahelpdesk.worldbank.org/knowledgebase/articles/906519-world-bank-country-and-lending-groups)). Note that this “gap” stresses broader issues: e.g., the importance of increased international funding for locally-led research in these geographies, literature reviews that include non-English-language literature and grey literature, and inclusive practices of international journals ([Pettorelli et al., 2021](https://besjournals.onlinelibrary.wiley.com/doi/epdf/10.1111/1365-2664.13815); [Nagendra, 2018](https://www.nature.com/articles/d41586-018-05210-0); [Nuñez et al., 2019](https://besjournals.onlinelibrary.wiley.com/doi/full/10.1111/1365-2664.13319); [Campos-Arceiz et al., 2018](https://doi.org/10.1016/j.biocon.2017.07.028); [Mammides et al., 2016](https://doi.org/10.1016/j.biocon.2016.03.030); [Habel et al., 2017](https://doi.org/10.1007/s10531-016-1236-1)). |
|  | Freshwater, coastal, and marine ecosystems. |
|  | Habitats in urban systems. |
|  | Assessment of multiple scales and smaller spatial scales (in addition to regional scales — e.g., local) to increase relevance to decision-making. |
| (ii) Ecosystem goods and services | Climate impacts on cultural ecosystem services. |
|  | Climate impacts on understudied ecosystem services such as medicinal resources, local climate and air quality, pollination, and biological control. |
|  | Climate impacts on ecosystem service delivery/demand (especially when assessed through stakeholder/rightsholder engagement) or monetary value. |
| (iii-iv) Climate and non-climate drivers | Assessment of observed climate impacts on ecosystem services, in addition to projected climate impacts. |
|  | Cumulative and individual assessment of multiple climate drivers to discern their relative influence. |
|  | Interactions between climate change and understudied non-climate drivers (i.e., those besides land use change). |
| (v) Uncertainty | Understudied, decision-relevant sources of uncertainty. E.g., uncertainty in how ecosystem services are delivered or demanded, how ecosystem services are supplied, or how climate change impacts ecosystem services. |
|  | Underused, decision-relevant methods for assessing uncertainty. E.g., multiple models for assessing uncertainty in how ecosystem services are supplied [(Willcock et al., 2020;](https://doi.org/10.1016/j.scitotenv.2020.141006) [Bryant et al., 2018](https://doi.org/10.1016/j.ecoser.2018.09.001)[)](https://doi.org/10.1016/j.scitotenv.2020.141006) or probabilistic approaches. |
| (vi) Decision-making | Effectively incorporating multiple types of uncertainty into decision-making. |

**References**

Bryant, B. P., Borsuk, M. E., Hamel, P., Oleson, K. L. L., Schulp, C. J. E., & Willcock, S. (2018). Transparent and feasible uncertainty assessment adds value to applied ecosystem services modeling. *Ecosystem Services*, *33*, 103–109.<https://doi.org/10.1016/j.ecoser.2018.09.001>

Campos-Arceiz, A., Primack, R. B., Miller-Rushing, A. J., & Maron, M. (2018). Striking underrepresentation of biodiversity-rich regions among editors of conservation journals. *Biological Conservation*, *220*, 330–333. <https://doi.org/10.1016/j.biocon.2017.07.028>

Chausson, A., Turner, B., Seddon, D., Chabaneix, N., Girardin, C. A. J., Kapos, V., Key, I., Roe, D., Smith, A., Woroniecki, S., & Seddon, N. (2020). Mapping the effectiveness of nature‐based solutions for climate change adaptation. *Global Change Biology*, *26*(11), 6134–6155. <https://doi.org/10.1111/gcb.15310>

Habel, J. C., Lens, L., Eggermont, H., Githiru, M., Mulwa, R. K., Shauri, H. S., Lewinsohn, T. M., Weisser, W. W., & Schmitt, T. (2017). More topics from the tropics: Additional thoughts to Mammides et al. *Biodiversity and Conservation*, *26*(1), 237–241. <https://doi.org/10.1007/s10531-016-1236-1>

Mammides, C., Goodale, U. M., Corlett, R. T., Chen, J., Bawa, K. S., Hariya, H., Jarrad, F., Primack, R. B., Ewing, H., Xia, X., & Goodale, E. (2016). Increasing geographic diversity in the international conservation literature: A stalled process? *Biological Conservation*, *198*, 78–83. <https://doi.org/10.1016/j.biocon.2016.03.030>

Nagendra, H. (2018). The global south is rich in sustainability lessons that students deserve to hear. *Nature*, *557*(7706), 485–488. <https://doi.org/10.1038/d41586-018-05210-0>

Nuñez, M. A., & Amano, T. (2021). Monolingual searches can limit and bias results in global literature reviews. *Nature Ecology & Evolution*, *5*(3), 264–264. <https://doi.org/10.1038/s41559-020-01369-w>

Pettorelli, N., Barlow, J., Nuñez, M. A., Rader, R., Stephens, P. A., Pinfield, T., & Newton, E. (2021). How international journals can support ecology from the Global South. *Journal of Applied Ecology*, *58*(1), 4–8. <https://doi.org/10.1111/1365-2664.13815>

Runting, R. K., Bryan, B. A., Dee, L. E., Maseyk, F. J. F., Mandle, L., Hamel, P., Wilson, K. A., Yetka, K., Possingham, H. P., & Rhodes, J. R. (2017). Incorporating climate change into ecosystem service assessments and decisions: A review. *Global Change Biology*, *23*(1), 28–41.<https://doi.org/10.1111/gcb.13457>

Willcock, S., Hooftman, D. A. P., Blanchard, R., Dawson, T. P., Hickler, T., Lindeskog, M., Martinez-Lopez, J., Reyers, B., Watts, S. M., Eigenbrod, F., & Bullock, J. M. (2020). Ensembles of ecosystem service models can improve accuracy and indicate uncertainty. *Science of The Total Environment*, *747*, 141006. <https://doi.org/10.1016/j.scitotenv.2020.141006>

World Bank Country and Lending Groups – World Bank Data Help Desk [cited 25 February 2024]. Available from<https://datahelpdesk.worldbank.org/knowledgebase/articles/906519-world-bank-country-and-lending-groups>
