# Supplementary figures and images for: Climate change effects on ecosystem services: Disentangling drivers of mixed responses
Source: PLoS One. 2025 Feb 10;20(2):e0306017. doi: 10.1371/journal.pone.0306017 (PMC11809903; doi:10.1371/journal.pone.0306017)

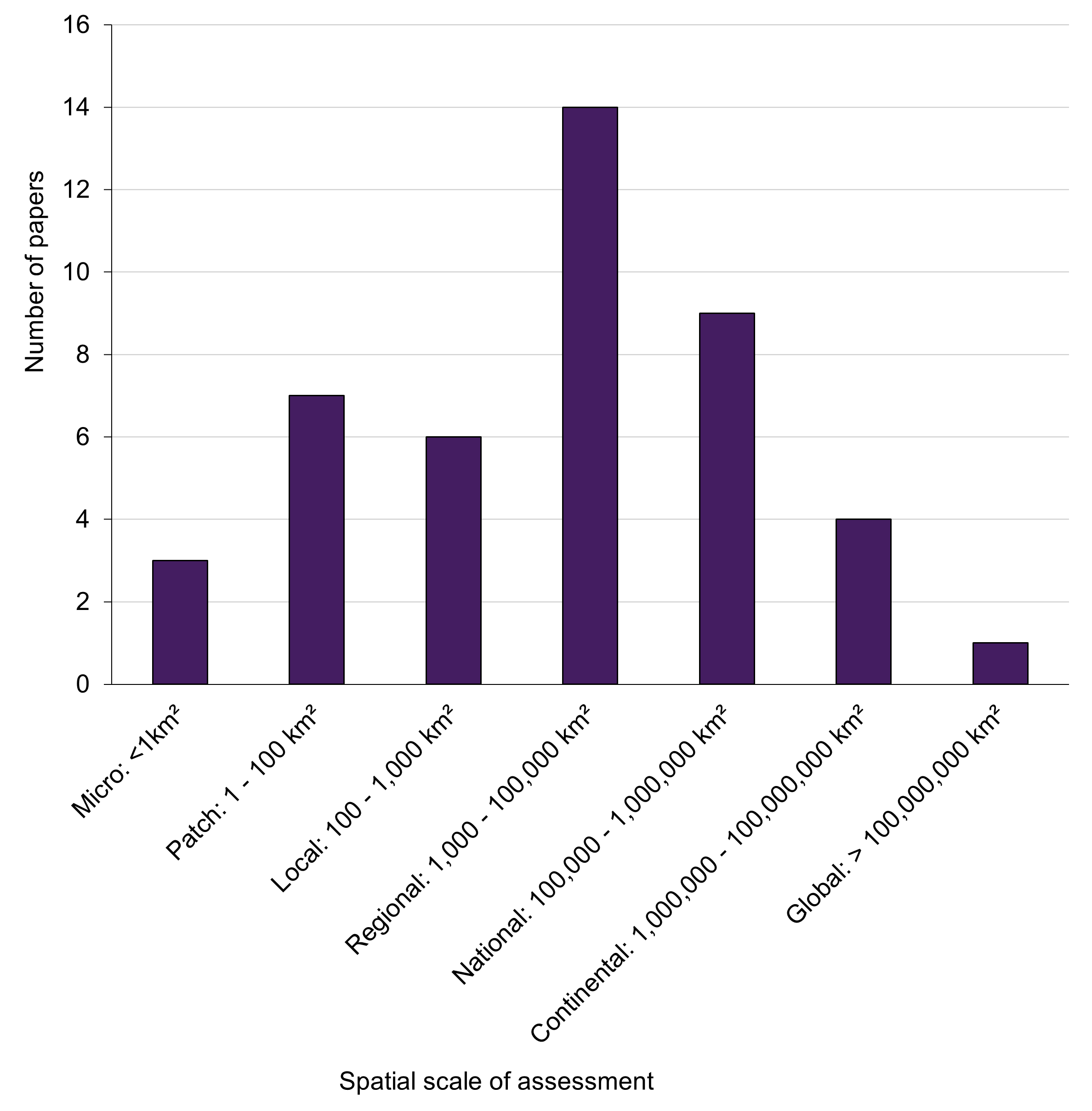

Supplement: S1 Fig — Categories include micro (<1 km2), patch (1–100 km2), local (100–1,000 km2), regional (1,000–100,000 km2), national (100,000–1,000,000 km2), continental (1,000,000–100,000,000 km2), and global (>100,000,000 km2). The spatial scale recorded per study was the largest scale at which climate impacts on ecosystem services were assessed. N = 44 papers. (TIF) [file pone.0306017.s006.tif]

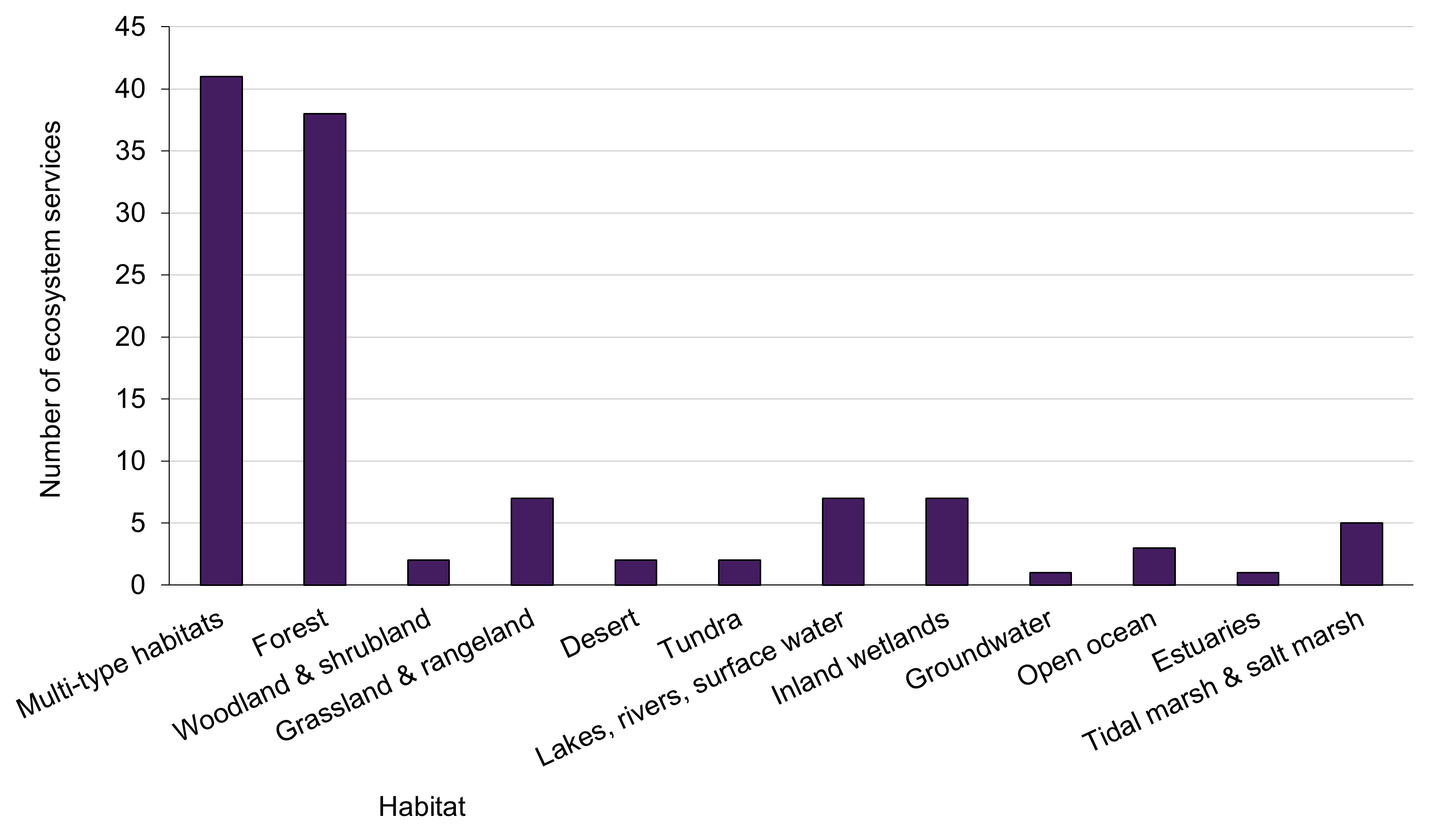

Supplement: S2 Fig — N = 116 services assessed for climate impacts in habitats or time periods. (TIF) [file pone.0306017.s007.tif]

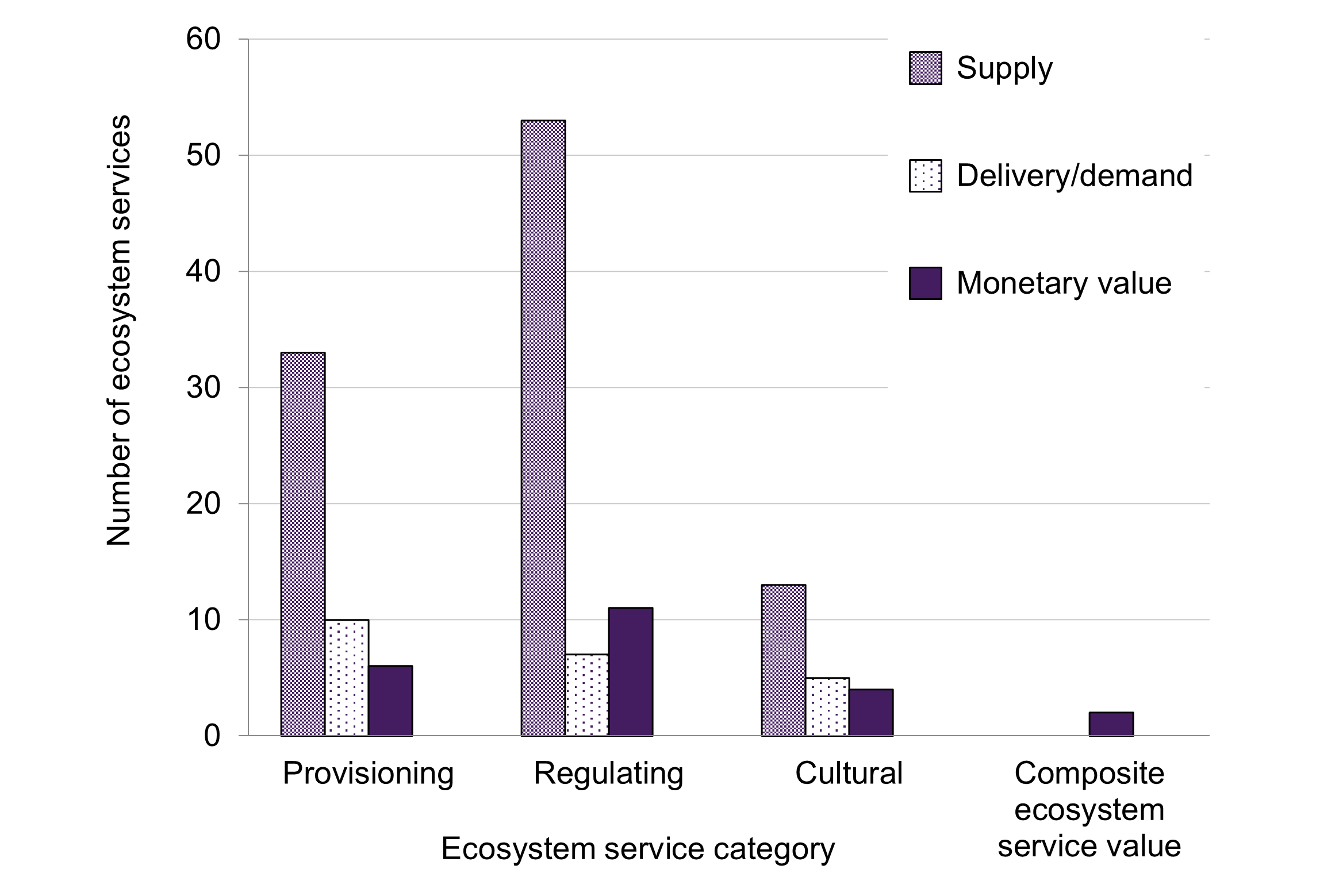

Supplement: S3 Fig — N = 144 EGS supply, delivery/demand, or monetary value responses to climate impacts in specific habitats and time periods. (TIF) [file pone.0306017.s008.tif]

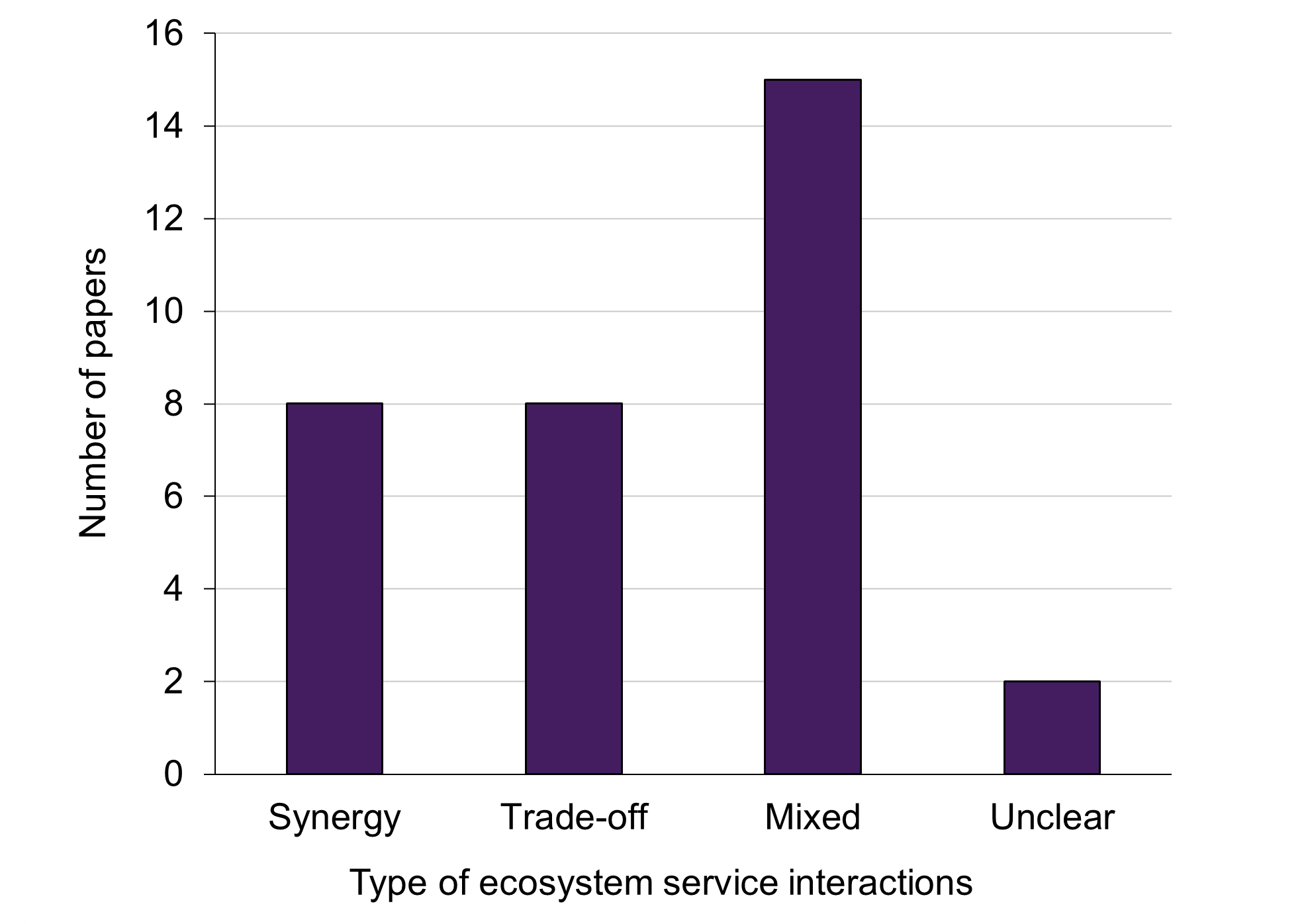

Supplement: S4 Fig — Interactions could be between a provisioning, regulating, or cultural service and supporting services (e.g., biodiversity or habitat extent). A paper could assess multiple service interaction types (e.g., if the paper had multiple services with interactions each assessed). Consequently, the total number of ecosystem service interactions (33) summed to more than the total number of papers that clearly assessed interactions (31). (TIF) [file pone.0306017.s009.tif]

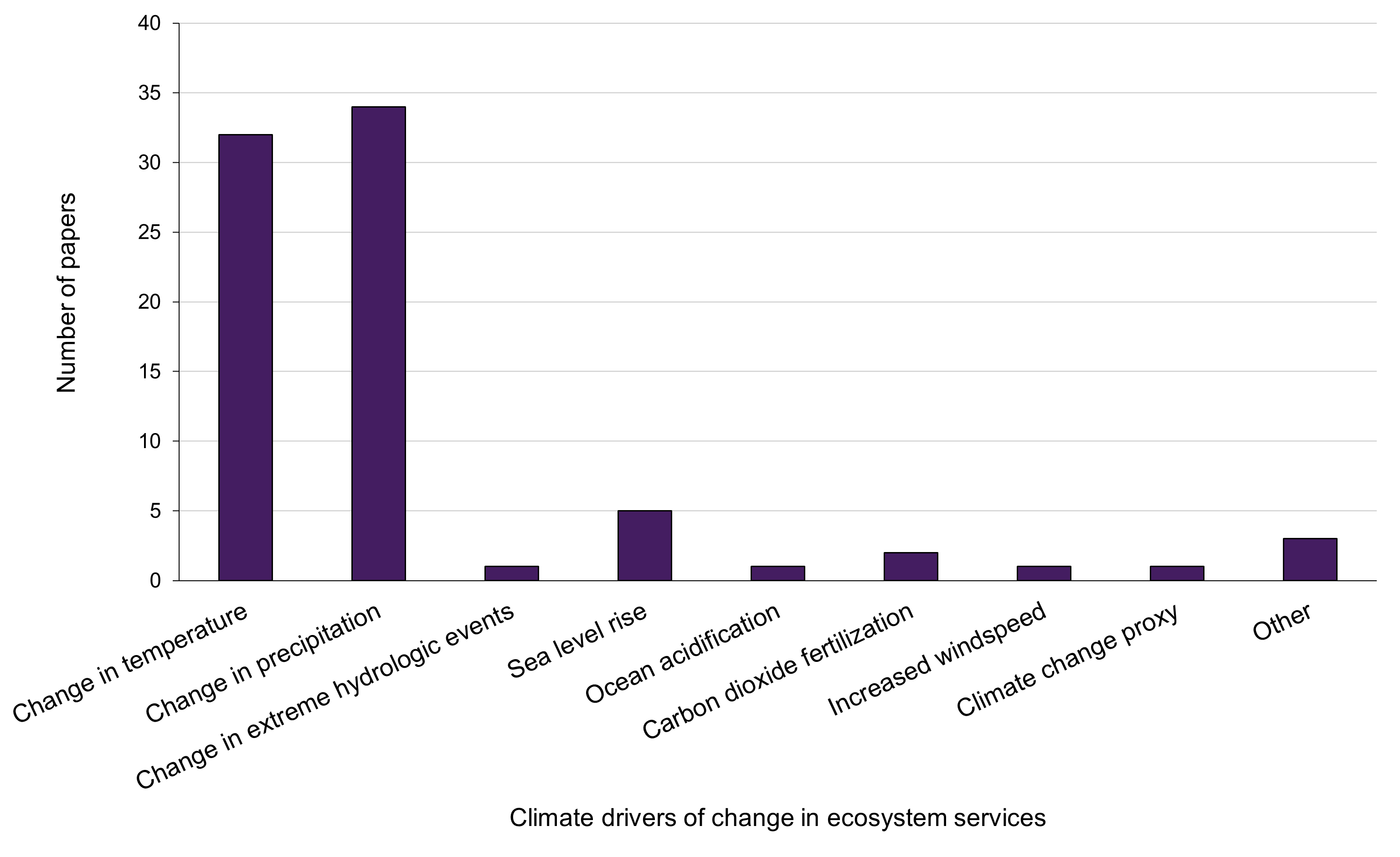

Supplement: S5 Fig — Since a paper could assess impacts of multiple climate drivers (an approach of 32 papers), the total number of climate drivers at the paper-level (80) is greater than the total number of papers (44). (TIF) [file pone.0306017.s010.tif]

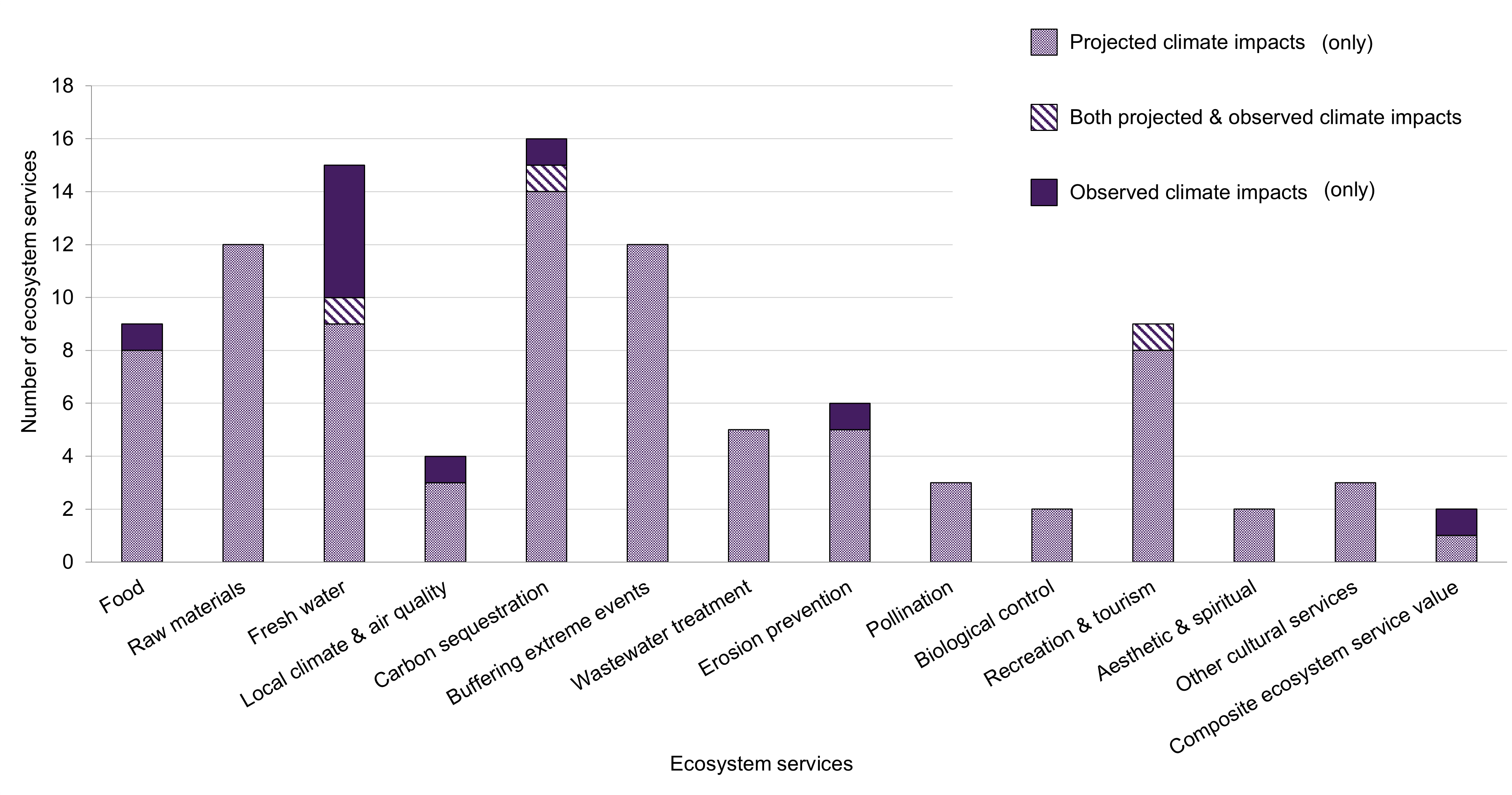

Supplement: S6 Fig — N = 100 ecosystem services assessed within papers, without incorporating additional counts of services assessed in specific habitats or time periods. (TIF) [file pone.0306017.s011.tif]

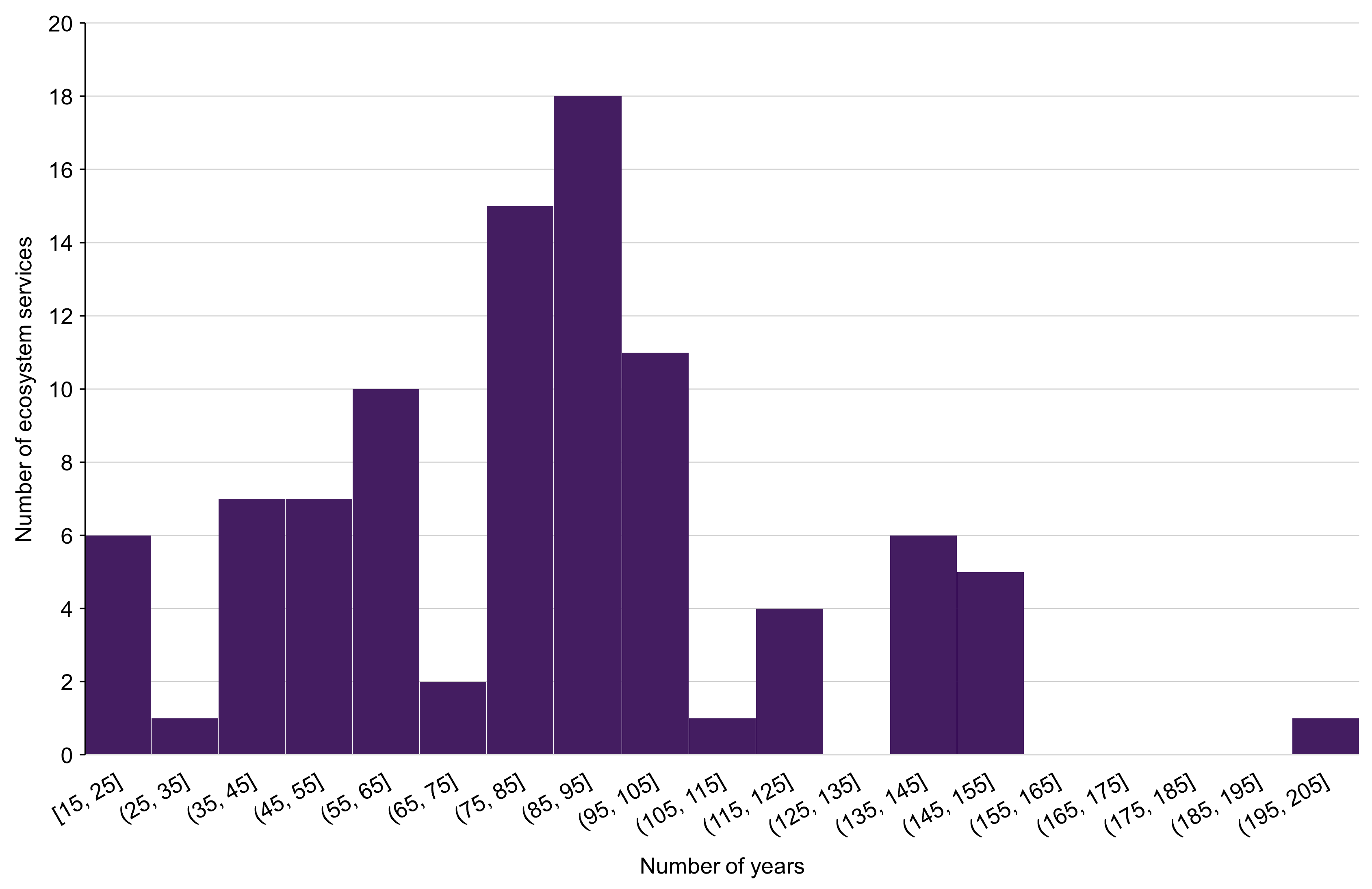

Supplement: S7 Fig — N = 94 services (81% of the total 116 services assessed by habitat or time period, since the total number of years within the remaining 22 services were either not assessed or unclear). Each range of years is inclusive of the bracketed numbers shown in the figure. (TIF) [file pone.0306017.s012.tif]

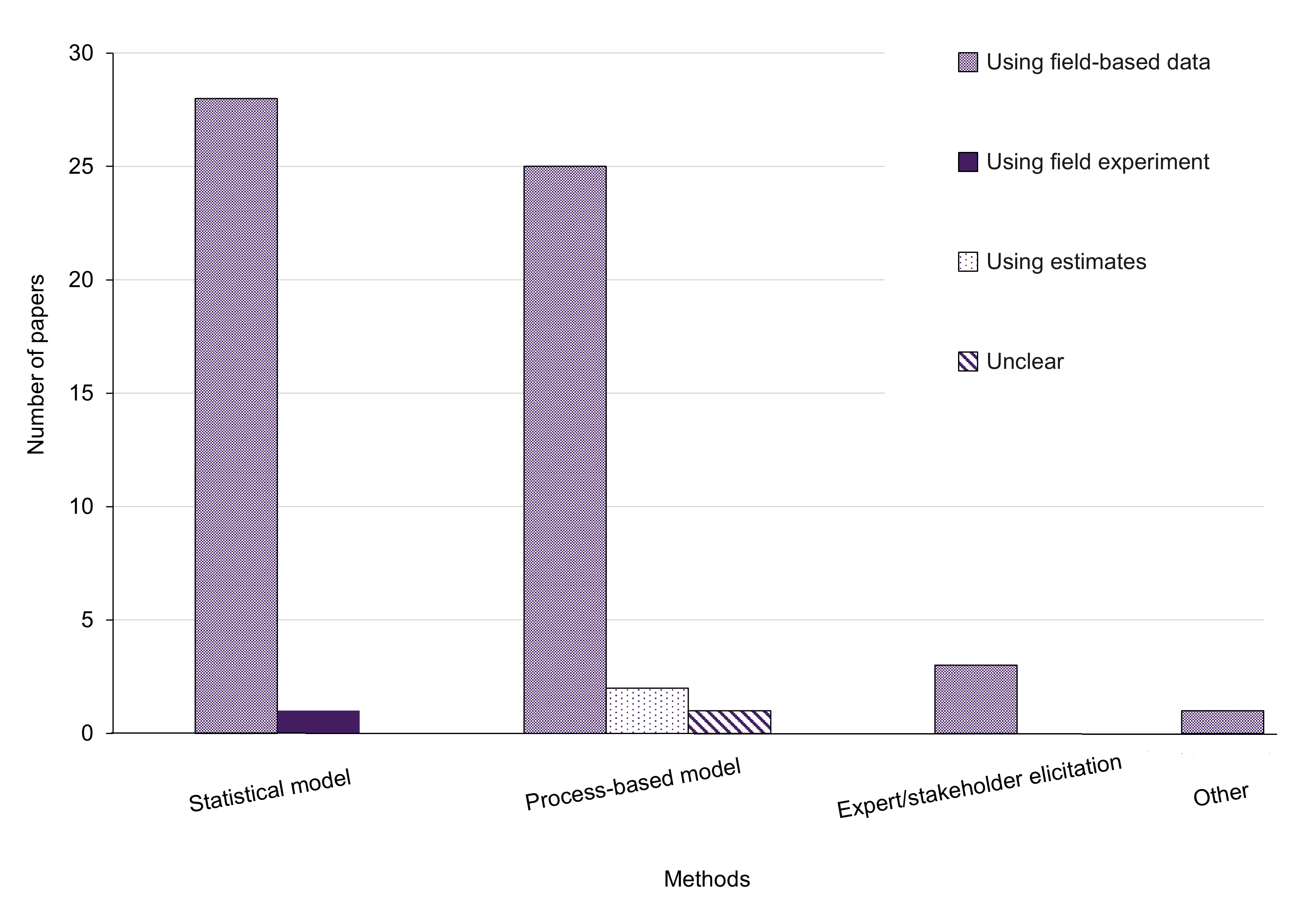

Supplement: S8 Fig — A paper could incorporate multiple methods to link climate impacts to services; therefore, the total number of methods at the paper-level (61) is greater than the total number of papers (44). (TIF) [file pone.0306017.s013.tif]

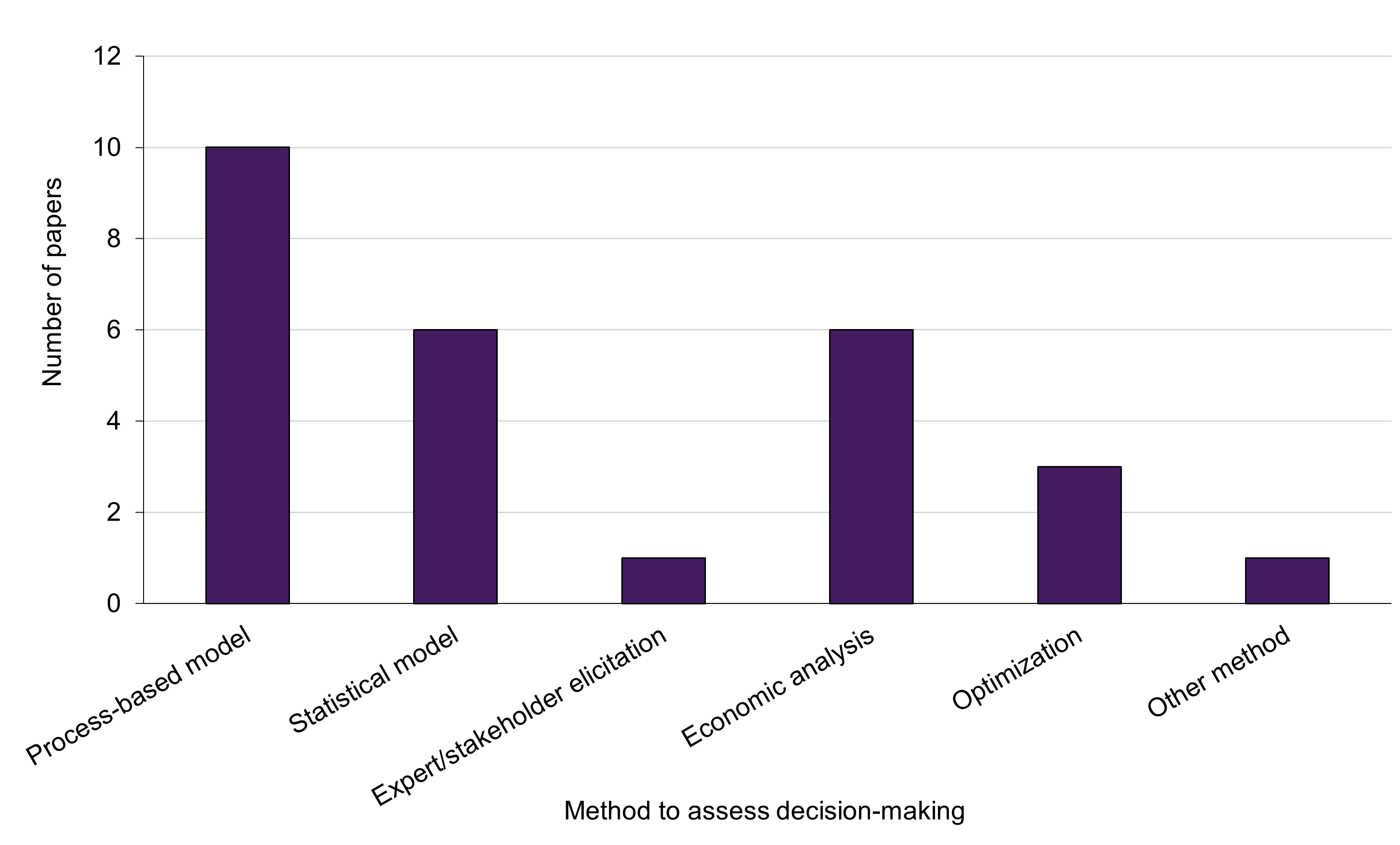

Supplement: S9 Fig — A paper could incorporate multiple methods to assess decision-making. This caused the total number of methods to assess decision-making at the paper-level (24) to be greater than the total number of papers that assessed decision-making (19). (TIF) [file pone.0306017.s014.tif]
